# Derivation of adult canine intestinal organoids for translational research in gastroenterology

**DOI:** 10.1186/s12915-019-0652-6

**Published:** 2019-04-11

**Authors:** Lawrance Chandra, Dana C. Borcherding, Dawn Kingsbury, Todd Atherly, Yoko M. Ambrosini, Agnes Bourgois-Mochel, Wang Yuan, Michael Kimber, Yijun Qi, Qun Wang, Michael Wannemuehler, N. Matthew Ellinwood, Elizabeth Snella, Martin Martin, Melissa Skala, David Meyerholz, Mary Estes, Martin E. Fernandez-Zapico, Albert E. Jergens, Jonathan P. Mochel, Karin Allenspach

**Affiliations:** 1 Departments of Veterinary Clinical Sciences, Iowa State University, Ames, IA USA; 2 Biomedical Sciences, Iowa State University, Ames, IA USA; 3 Departments of Chemical and Biological Engineering, Iowa State University, Ames, IA USA; 4 Veterinary Microbiology and Preventative Medicine, College of Veterinary Medicine, Iowa State University, Ames, IA USA; 5 Animal Science, Iowa State University, Ames, IA USA; 6 UCLA School of Medicine, Los Angeles, CA USA; 7 Biomedical Engineering, University of Wisconsin, Madison, WI USA; 8 Division of Comparative Pathology, University of Iowa Carver College of Medicine, Iowa City, USA; 9 Baylor College of Medicine, Houston, TX USA; 10 Schulze Center for Novel Therapeutics, Division of Oncology Research, Mayo Clinic, Rochester, MN USA

**Keywords:** Organoid model, Canine, Enteroid, GI diseases, Translational research, Intestinal stem cell

## Abstract

**Background:**

Large animal models, such as the dog, are increasingly being used for studying diseases including gastrointestinal (GI) disorders. Dogs share similar environmental, genomic, anatomical, and intestinal physiologic features with humans. To bridge the gap between commonly used animal models, such as rodents, and humans, and expand the translational potential of the dog model, we developed a three-dimensional (3D) canine GI organoid (enteroid and colonoid) system. Organoids have recently gained interest in translational research as this model system better recapitulates the physiological and molecular features of the tissue environment in comparison with two-dimensional cultures.

**Results:**

Organoids were derived from tissue of more than 40 healthy dogs and dogs with GI conditions, including inflammatory bowel disease (IBD) and intestinal carcinomas. Adult intestinal stem cells (ISC) were isolated from whole jejunal tissue as well as endoscopically obtained duodenal, ileal, and colonic biopsy samples using an optimized culture protocol. Intestinal organoids were comprehensively characterized using histology, immunohistochemistry, RNA in situ hybridization, and transmission electron microscopy, to determine the extent to which they recapitulated the in vivo tissue characteristics. Physiological relevance of the enteroid system was defined using functional assays such as optical metabolic imaging (OMI), the cystic fibrosis transmembrane conductance regulator (CFTR) function assay, and Exosome-Like Vesicles (EV) uptake assay, as a basis for wider applications of this technology in basic, preclinical and translational GI research. We have furthermore created a collection of cryopreserved organoids to facilitate future research.

**Conclusions:**

We establish the canine GI organoid systems as a model to study naturally occurring intestinal diseases in dogs and humans, and that can be used for toxicology studies, for analysis of host-pathogen interactions, and for other translational applications.

**Electronic supplementary material:**

The online version of this article (10.1186/s12915-019-0652-6) contains supplementary material, which is available to authorized users.

## Background

Rodent models, especially the mouse, have been extensively used to study gastrointestinal (GI) diseases due to cost effectiveness, ethical considerations, and the easy accessibility to genetically engineered technology. Despite the wide use of mouse models in biomedical research, the translational value of mouse studies for human disease remains controversial [1]. In addition, mice and other rodents often fail to adequately represent the human condition, as well as drug response in toxicity and efficacy studies [2, 3]. Given the high failure rate of drugs from discovery and development through the clinical trial phase (i.e., more than 90%), there is now a critical need for better animal models for preclinical studies [4].

Large animal models, such as the dog, are typically more representative than mice as they have a relatively large body size, longer life span, more closely resemble human GI physiology, and develop spontaneous, analogous diseases including inflammatory bowel disease (IBD) and colorectal cancer (CRC) [4]. Dogs have been used as an animal model for human health and disease from the ancient to the modern era [5, 6]. The dog is still considered to be a superior non-rodent mammalian animal model for pharmaceutical research and is preferred by the FDA for initial safety data of drugs for human use [7]. Although the dog has contributed immensely to the advancement of medical knowledge in the past, the use of the dog in medical research has declined in recent years due to the emotional perceptions among the public and ensuing ethical issues with canine research [5]. The canine GI organoids arose as a model to bridge the gap in the drug development pipeline by providing a more representative in vitro model to test drug efficacy and toxicity in preclinical studies, as well as an innovative screening tool in drug discovery, while also reducing the number of animals needed for in vivo studies [2, 4, 8]. Thus, the ultimate goal of our research is to culture canine intestinal organoids from diseased dogs to develop better therapeutic strategies and personalized medicine for both animal and human health.

Stem cell-derived 3D organoids have emerged as a cutting edge cell culture technology to study the developmental biology of the intestines, brain, stomach, and liver [9–12]; drug discovery and toxicity screening [4]; drug testing for personalized medicine [4, 13]; infectious disease biology of viruses [14, 15]; and regenerative medicine [16]. Organoids are collections of organ-specific cell aggregates derived from either primary tissue or stem cells that are capable of organ-like functionality in an in vitro environment [17, 18]. The 3D organoid model better reproduces the in vivo biology, structure, and function, as well as genetic and epigenetic signatures of original tissues, unlike widely used two-dimensional (2D) cell monolayer models that utilize cancer and immortalized cell lines [4, 19–21].

Organoids are developed from either embryonic or induced pluripotent mesenchymal-derived stem cells (iPSC) or organ-specific adult stem cells (ASC) [17, 19]. Organoids derived from ASCs are generated without genetic transduction by transcription factors, unlike organoids derived from iPSCs [19], thus providing a more physiologically relevant in vitro model than iPSC-derived organoids. ASC-derived organoids are a functional model that can be differentiated to replicate the in vivo adult environment and can be safely transplanted into animals and humans [22, 23]. In addition, adult intestinal stem cell (ISC)-derived organoids have recently gained attention as a model to understand how the intestinal epithelia interact with the gut microbiome to modulate GI health and disease, for the study of infectious diseases of the GI tract, and as a drug screening tool for personalized medicine in diseases such as cystic fibrosis (CF) [13, 24].

In this study, we have developed 3D canine intestinal organoids from a large number of healthy dogs and dogs with GI diseases, including IBD and intestinal carcinomas. Intestinal organoids, propagated from leucine-rich repeat-containing G protein-coupled receptor 5 (Lgr5)-positive stem cells located in intestinal crypts, are termed “enteroids” or “colonoids,” depending on the anatomic region of origin (i.e., small vs. large intestine). Jejunal 3D enteroids were characterized from healthy dogs using histopathology, immunohistochemistry (IHC), RNA in situ hybridization (RNA-ISH), and transmission electron microscopy (TEM) and compared to whole jejunal tissues to prove the reproducibility and translatability of this in vitro model. The physiological relevance of the canine enteroid system was further demonstrated using functional assays, including optical metabolic imaging (OMI), the cystic fibrosis transmembrane conductance regulator (CFTR) function assay, and the Exosome-Like Vesicles (ELVs) uptake assay. In summary, 3D canine enteroids are a relevant in vitro animal model with wide applications in veterinary and translational biomedical research: (1) to perform mechanistic studies for basic GI research; (2) for applied preclinical drug permeability, efficacy, and safety testing; (3) for personalized medicine in animal health; and (4) for preclinical research prior to in vivo clinical trials in human patients.

## Results

### Development of 3D cultures of canine enteroids and colonoids

**Fig. 1:**
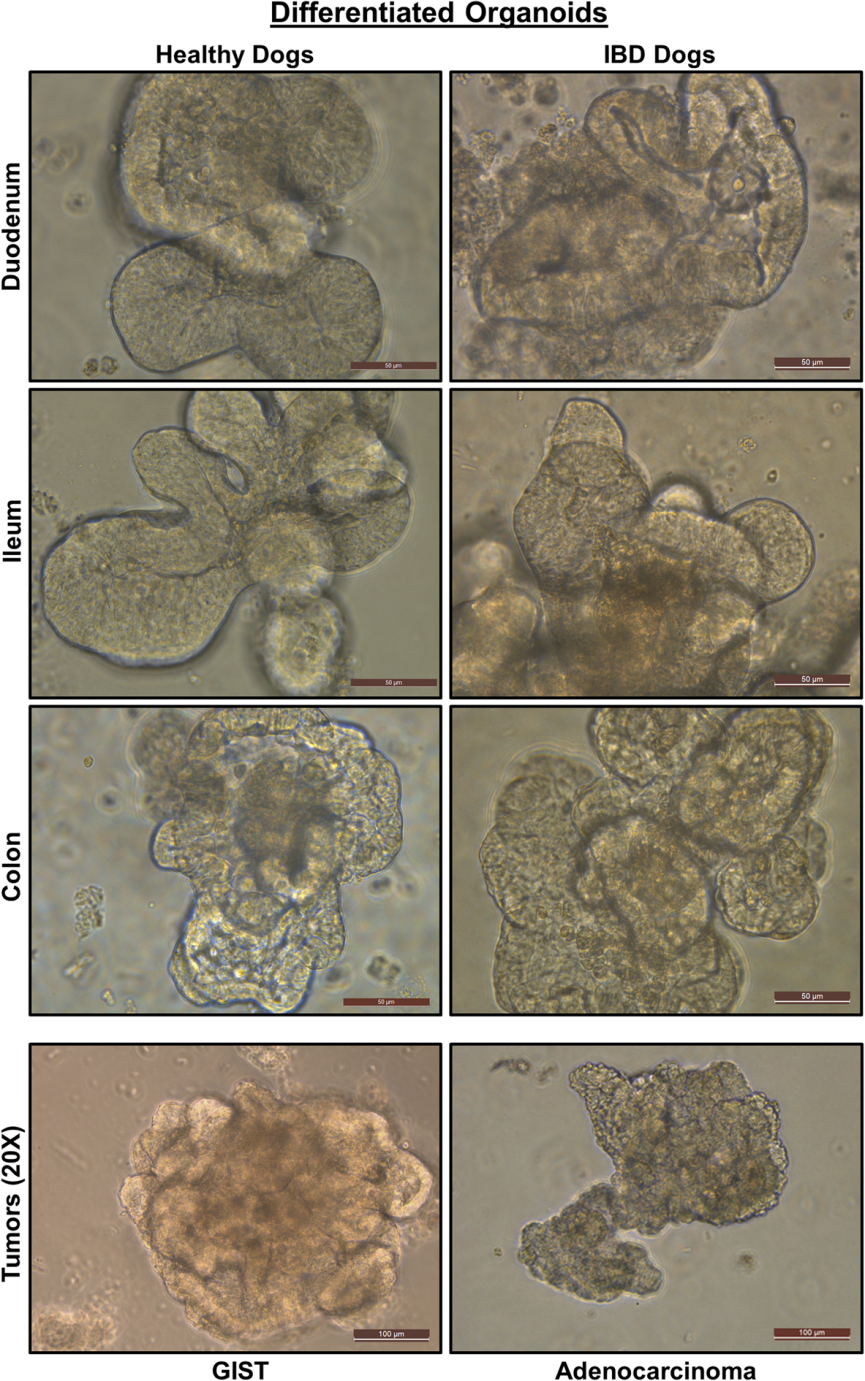
Canine intestinal 3D organoids (enteroids and colonoids) from healthy and inflammatory bowel disease (IBD) dogs. Representative phase contrast images at × 40 magnification of fully differentiated 5–7-day-old duodenal and ileal enteroids and colonoids from healthy dogs and dogs with IBD, as well as gastrointestinal stromal tumor (GIST) and colorectal adenocarcinoma tumor (tumors at × 20 magnification). The structures of enteroids and colonoids from healthy dogs and dogs with IBD appeared comparable. Representative photomicrographs of at least *n* = 15 (healthy dogs), *n* = 7 (IBD dogs), and *n* = 4 (tumors) images of enteroids/colonoids are shown for each location as indicated

Using isolated canine intestine crypts, which contain adult intestinal stem cells (ISC) at the crypt base, we developed 3D intestinal enteroids and colonoids from 28 healthy and 12 diseased dogs, including dogs diagnosed with IBD (*N* = 9) and intestinal tumors (*N* = 2). Enteroids and colonoids were collected from different intestinal segments, including the duodenum, jejunum, ileum, and colon, as well as from intestinal tumors, and were maintained for up to over 20 passages. A summary of the demographics of dogs used for canine intestinal stem cell isolation, culture, and maintenance is presented in Additional file 1: Table S1. Intestinal stem cell isolation and enteroid and colonoid maintenance followed a modified version of the procedure previously described by Saxena et al for human organoids, and the defined media included Wnt3a, R-spondin-1, and Noggin, as well as inhibitors of Rock and GSK3β for the first 2–3 days of culture [25]. Figure 1 reveals the phase contrast images of fully differentiated 5–7-day-old duodenal and ileal enteroids as well as colonoids from healthy dogs and dogs with IBD. We also show two intestinal tumors, a gastrointestinal stromal tumor (GIST) and colorectal adenocarcinoma tumor (Fig. 1, bottom panels). There were no major morphological differences in the structures of enteroids and colonoids from healthy dogs and dogs with IBD, as visualized by bright field microscopy. We created a bio-archive of cryopreserved enteroids and colonoids for future use in basic and applied research. This resource, which is to-date the largest collection of 3D enteroids and colonoids from healthy and diseased dogs, will be made available to the scientific community (Additional file 1: Table S1).

### Morphologic characterization of canine organoids

**Fig. 2:**
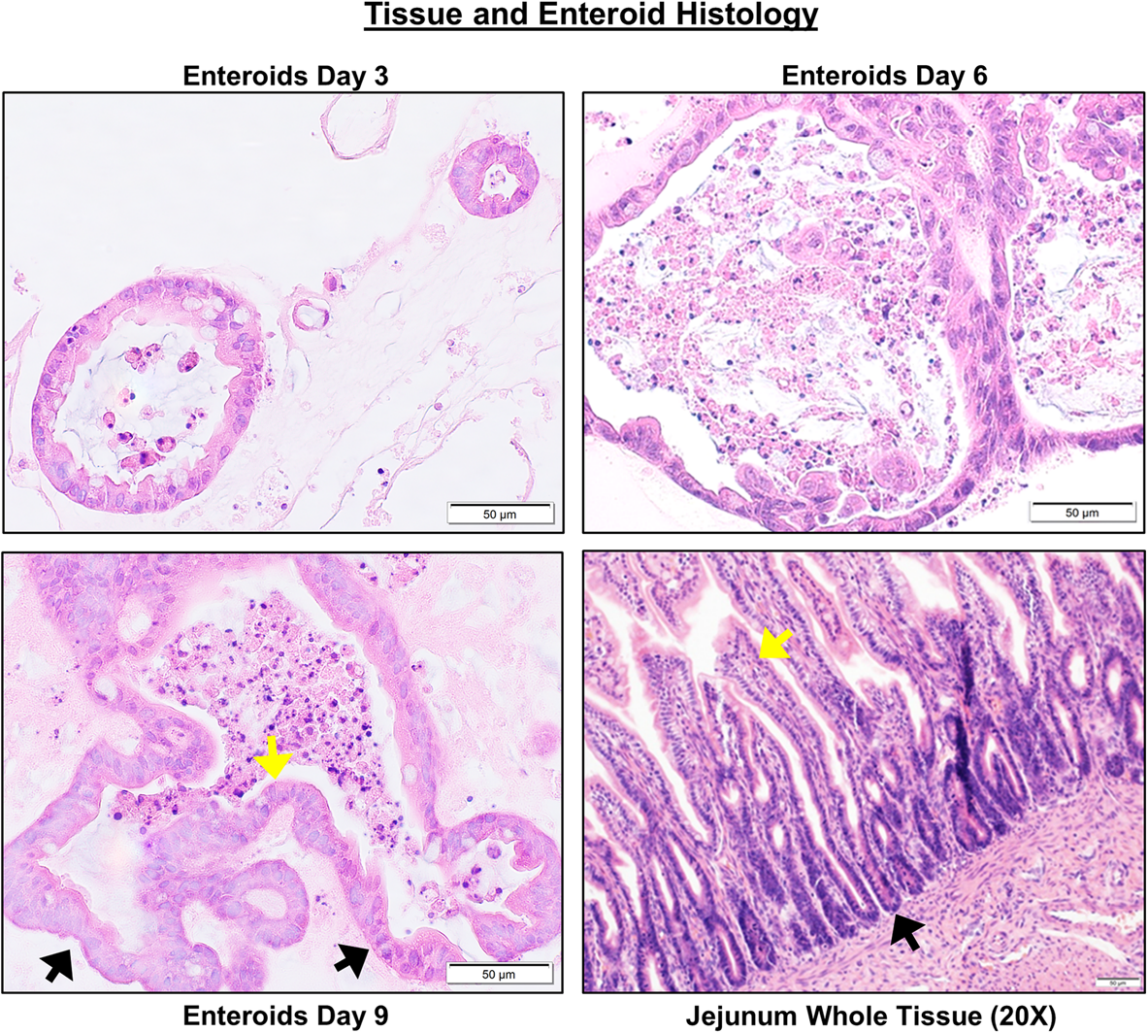
Characterization of canine jejunal tissue and jejunal enteroid histology shows similarities of epithelial structure. Histological images of hematoxylin and eosin (H&E) staining show the development and differentiation of canine enteroids at 3, 6, and 9 days after isolation or passaging. Spheroid-like epithelial structures are visualized in 3-day enteroids compared to crypt-villi epithelial structures on the sixth and ninth days. In the whole jejunal tissue, there are crypt-villi epithelial structures as well as non-epithelial cell types. Arrows indicate examples of crypts (black arrows) and villi (yellow arrows). Representative images from at least *n* = 15 enteroids per condition

Histology of enteroids on days 3, 6, and 9 of differentiation is shown in representative images of H&E tissue sections (Fig. 2). Day 3 enteroids have undifferentiated cyst-like structures embedded in pink-colored Matrigel 3D matrix, whereas days 6 and 9 enteroids present both crypt and villi-like structures embedded in Matrigel. Dark purple staining bodies in the enteroid lumen represent epithelial cells that have undergone apoptosis in day 9 tissue sections. For comparison, the H&E tissue sections of jejunal mucosa reveal normal tissue architecture.

**Fig. 3:**
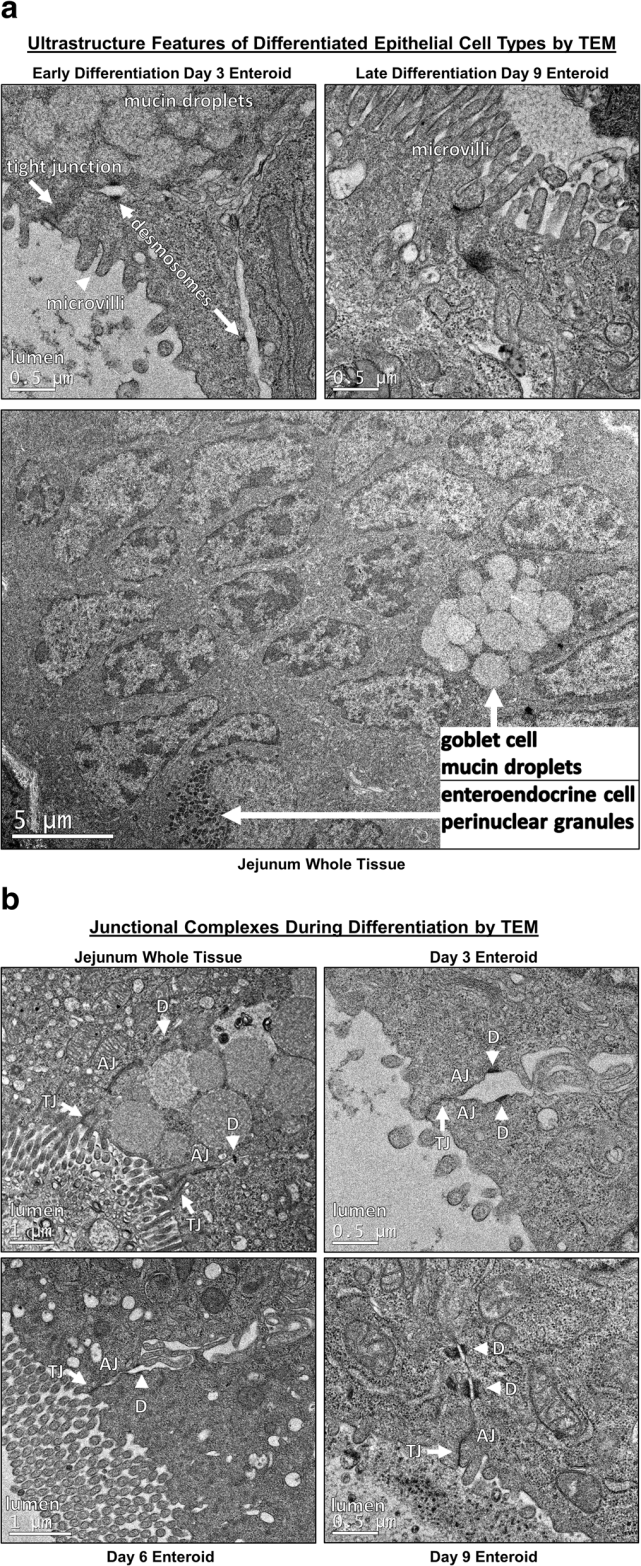
Ultrastructural features of differentiated enteroids mimic intact small intestinal tissue. **a** Canine jejunum enteroids, during early (day 3) and late (day 9) stages of differentiation, using whole tissue showing features of cellular differentiation, such as apical microvilli, electron-lucent cytoplasmic vacuoles (i.e., mucus), and electron-dense perinuclear granules (i.e., neurosecretory granules), consistent with development of absorptive enterocytes, goblet cells, and enteroendocrine cells, respectively. Ultrastructure features are visualized by representative transmission electron micrographs (TEM). **b** TEM of canine organoids show the progressive development (days 3–9 of differentiation) of intercellular structures important for intestinal barrier function. Adherens junction (AJ), tight junction (TJ), and desmosomes (D) structures are seen in both canine enteroids and native jejunum. On day 3, the developing tight junction had dilated paracellular space adjacent to tight junctions; however, the paracellular spaces were smaller on day 6, and no longer apparent by day 9. Representative images from at least *n* = 10 enteroids per condition

To identify changes in the ultrastructural and morphometric details of enteroids during differentiation on days 3, 6, and 9, we used TEM, which allowed longitudinal assessment of morphologic features. TEM revealed that differentiation of cells also occurs on an ultrastructural level. In jejunum tissue and enteroids, we visualized morphologic features of differentiated epithelial cells, including electron-lucent cytoplasmic vacuoles in mucus-producing goblet cells, and electron-dense perinuclear (neurosecretory) granules in enteroendocrine cells (Fig. 3a). In addition, TEM showed the presence of microvilli at the apical border of epithelial cells, which increased in length and number throughout enteroid differentiation from day 3 to 9 (Fig. 3a). Microvilli are cellular membrane protrusions of absorptive enterocytes containing different populations of brush border enzymes involved in absorption, secretion, and cellular adhesion [26]. Evidence of epithelial differentiation also involves increasingly adherent inter-epithelial structures, including adherens junctions (AJ), tight junctions (TJ), and desmosomes, and these were identified in both canine enteroids and in native jejunum (Fig. 3b). On day 3, the developing tight junction structure had a dilated paracellular space between epithelial cells, with the space diminishing on day 6 and no longer apparent by day 9 (Fig. 3b).

### Molecular marker analysis of canine organoids

**Fig. 4:**
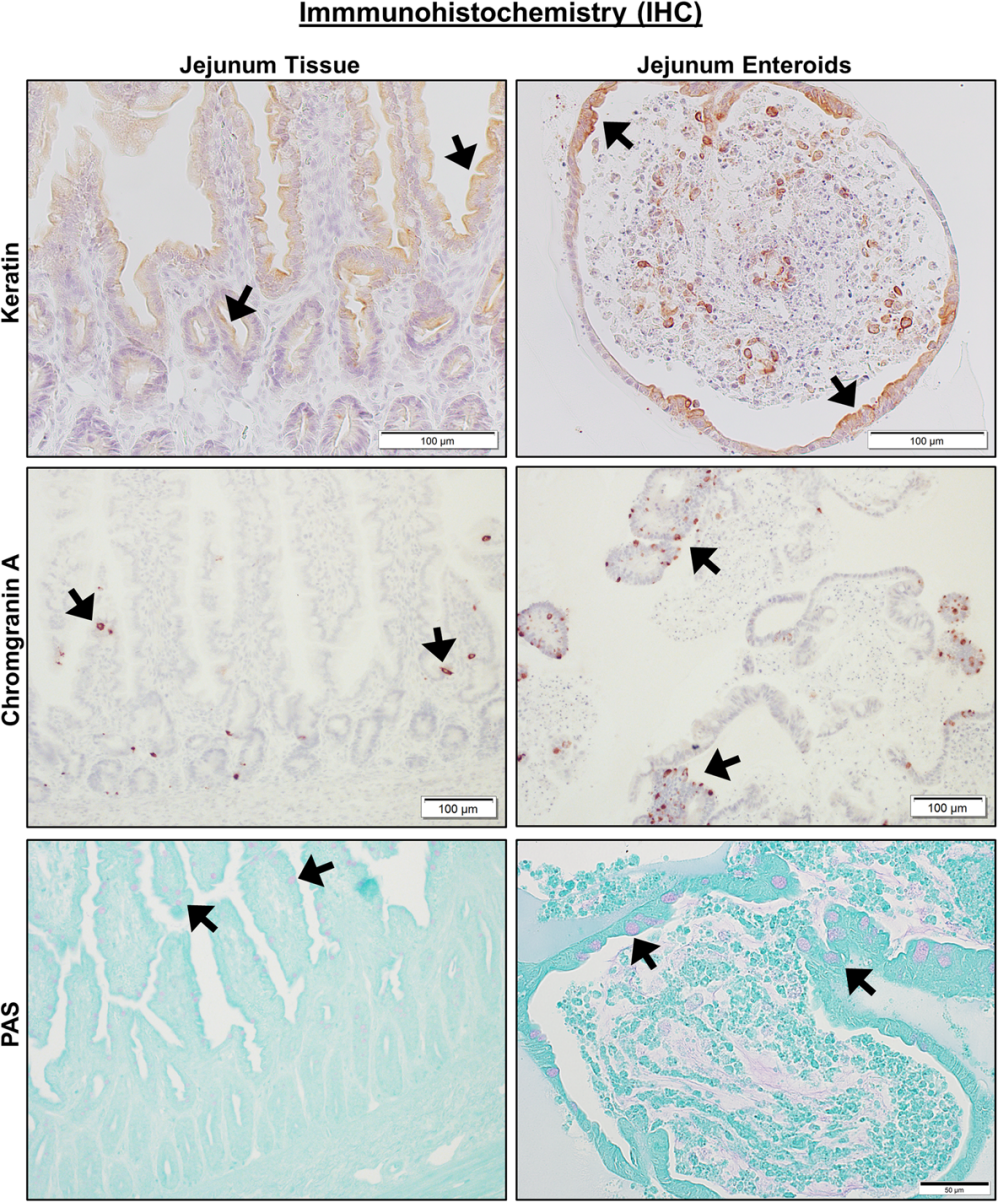
Canine jejunal tissues and enteroids both express markers of epithelial cell lineage. Representative immunohistochemistry (IHC) images comparing staining for marker proteins of epithelial cells and their lineage, including Keratin (epithelial cells, *upper panels*), Chromogranin A (enteroendocrine cells, *middle panels*), and PAS (goblet cells, *lower panels*) on both intact whole jejunal tissues and jejunal enteroids. Black arrows indicate representative positive staining for epithelial, enteroendocrine, or goblet cells. Tissue and enteroids were counterstained with Hematoxylin (*upper* and *middle panels*) or Alcian Blue (*lower panels*). Representative images from *n* = 20 or more enteroids per condition

We first performed phenotypic characterization of jejunal enteroids due to their importance in nutrient absorption and drug permeability. For IHC, we used antibodies to identify cell surface expression of epithelial cells and their lineage proteins (Pan-Keratin for epithelial cells, Chromogranin A for enteroendocrine cells, and PAS for goblet cells), mesenchymal cells (Vimentin and Actin), Paneth cells (Lysozyme), and immune cells (c-Kit and CD3) in jejunal enteroids compared to full-thickness jejunal tissues. IHC markers for epithelial cells and their lineage, such as Keratin, Chromogranin A, and PAS, had positive staining in both intact jejunal tissues and jejunal enteroids (Fig. 4 and Table 1). Keratin staining was absent in the lamina propria region of whole jejunal tissue, confirming the specificity of the epithelial marker in canine intestines [27]. Lysozyme, a marker of Paneth cells, was absent in both enteroids and jejunal epithelium (Table 1), which is consistent with previous reports showing that canine intestines lack Paneth cells [28]. Conversely and as expected, jejunal enteroids did not express markers for mesenchymal cells (vimentin and actin) or immune cells (c-Kit and CD3, a T cell marker) as they only contained epithelial cells (Table 1), whereas intact jejunal tissues robustly expressed all mesenchymal and immune cell markers in the lamina propria (Table 1).

**Table 1:** Characterization of canine jejunal tissues and jejunal enteroids using IHC

Tissue/enteroid	Stain/marker	Result	Location
Enteroid	Keratin/epithelium	Pos	Epithelium
Jejunum	Keratin/epithelium	Pos	Epithelium
Enteroid	PAS/goblet cell	Pos	Goblet cells
Jejunum	PAS/goblet cell	Pos	Goblet cells
Enteroid	Chromogranin A/enteroendocrine cell	Pos	Epithelium
Jejunum	Chromogranin A/enteroendocrine cell	Pos	Epithelium
Enteroid	Vimentin/mesenchymal cell	Neg	
Jejunum	Vimentin/mesenchymal cell	Pos	Submucosa, lamina propria vessels, muscularis
Enteroid	Actin/mesenchymal cell	Neg	
Jejunum	Actin/mesenchymal cell	Pos	Muscularis, lamina propria vessels
Enteroid	c-Kit/leukocyte	Neg	
Jejunum	c-Kit/leukocyte	Pos	Resident leukocytes
Enteroid	T cell/T lymphocyte	Neg	
Jejunum	T cell/T lymphocyte	Pos	Intraepithelial and lamina propria lymphocytes
Enteroid	Lysozyme/Paneth cell	Neg	Epithelium
Jejunum	Lysozyme/Paneth cell	Neg	Epithelium

### RNA in situ hybridization in enteroids

**Fig. 5:**
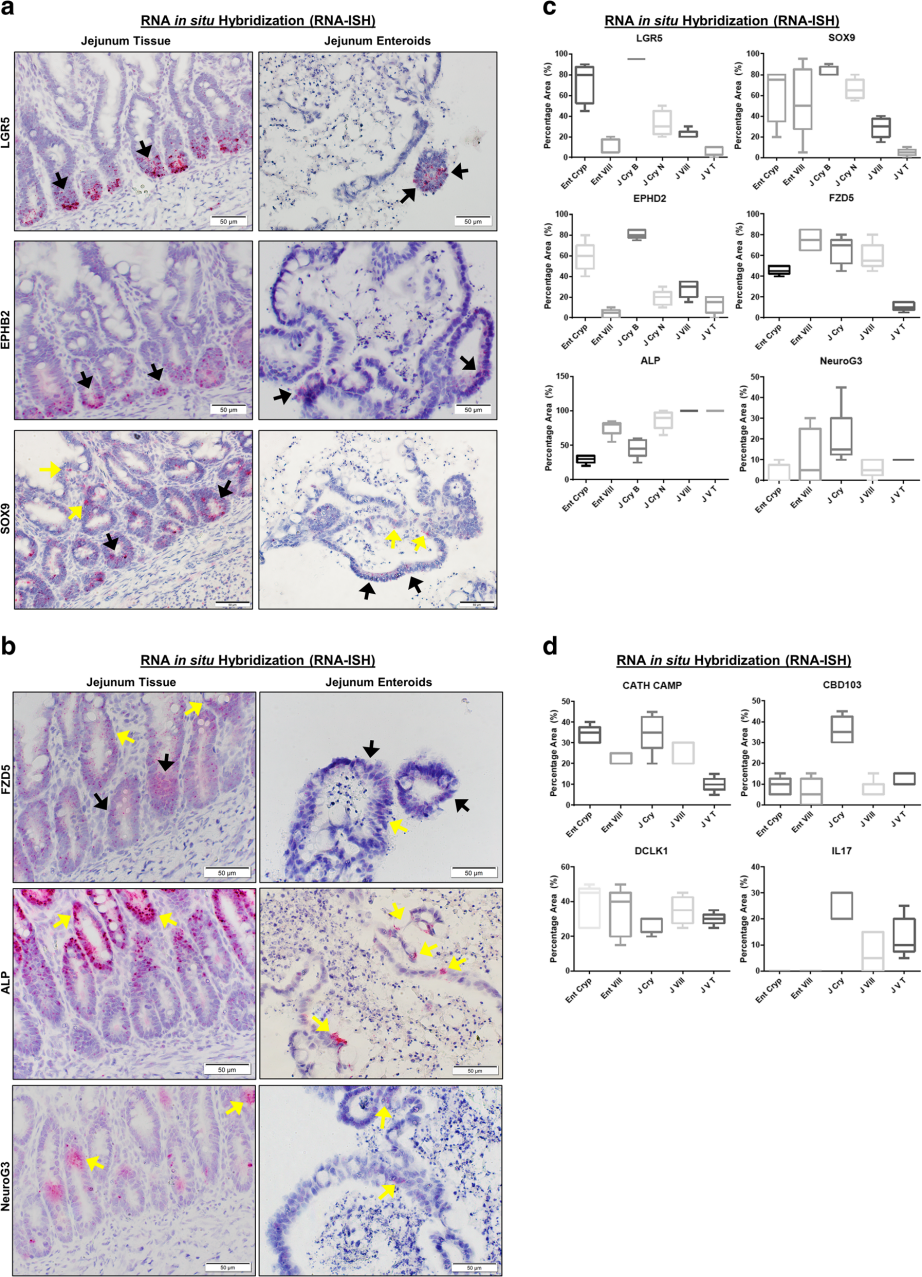
Both canine jejunal tissues and enteroids express mRNA for markers of various epithelial cell lineages. **a** Representative RNA in situ hybridization (RNA-ISH) images reveal expression of stem cell markers LGR5, SOX9, and EPHB2 on both intact jejunal tissues and enteroids. SOX9 is also a marker of enteroendocrine and tuft cells, while EPHB2 is also a marker of Paneth-like cells. Arrows indicate representative positive red areas in crypts (black arrows) or villi (yellow arrows). Representative images from at least *n* = 15 organoids per condition. **b** Representative images of gene expression for markers of epithelial lineage, including FZD5 (Paneth-like cell), ALP (absorptive epithelium), and Neuro G3 (Enteroendocrine cells) in both intact jejunal tissues and enteroids, as determined by RNA-ISH. Representative images from at least *n* = 15 organoids per condition. **c** Semi-quantitative scoring of RNA-ISH staining (box and whisker plots) for expression of stem cell markers (LGR5, SOX9), Paneth-like cell markers (FZD5, EPHB2), absorptive epithelial markers (ALP), and enteroendocrine cells (Neuro G3) in both intact jejunal tissues and enteroids. Specific sites include Enteroid Crypt (Ent Cryp), Enteroid Villus (Ent Vill), whole tissue Jejunum Crypt Base (J Cry B), Jejunum Crypt Neck (J Cry N), Jejunum Villus (J Vill), and Jejunum Villus Tip (J V T). Scoring of at least *n* = 6 images per condition. **d** Semi-quantitative expression of Paneth cell markers IL-17, CBD 103, and CATH as well as tuft cell marker Dclk1, in both intact jejunal tissues and enteroids, in specific sites as above. Cells and tissue were counterstained with hematoxylin. Scoring of at least *n* = 6 images per condition

Next, the RNA in situ hybridization (RNA-ISH) technology (RNAscope) was used to further characterize different intestinal stem cell populations secondary to epithelial cell differentiation, since there are no canine-specific antibodies available for many epithelial cell line-specific markers. RNAscope has a unique probe design strategy that allows for simultaneous signal amplification and background suppression to achieve single-molecule (mRNA) visualization, enabling identification of mRNA expression within cells and in the context of tissue architecture (Table 2) [29]. RNA-ISH shows spatial distribution and cell localization patterns of mRNA expressions, which is critical to identify cell types and their regional location, unlike quantitative PCR, which only provides aggregate mRNA expression. To study intestinal stem cell maturation, we used either early undifferentiated enteroids (3-day-old) or differentiated late enteroids (9-day-old). ISCs contained within enteroid crypts were identified with canine-specific probes for Leucine-rich repeat-containing G protein-coupled receptor 5 (LGR5) and SRY-related HMG-box sex-determining region Y (SRY)-related high-mobility group (HMG) box 9 (Sox9) [30–32]. LGR5, a seminal marker for adult intestinal stem cells [30, 31], was observed mainly in the enteroid crypt region of the whole jejunal crypt base (Fig. 5a) and quantitated (Fig. 5c). As expected, LGR5 expression was significantly reduced (*P* = 0.0001) in the villus portion of both enteroids and full-thickness jejunum (Fig. 5a, c) as compared to the crypt, confirming that canine intestinal stem cells reside mostly in the crypt area as described in other species [28]. Interestingly, SOX9, a marker for stem cell progenitors [33], was expressed in both crypts and villi of enteroids. However, jejunal crypts had significantly greater SOX9 expression than that observed in their corresponding villi (*P* = 0.0021), indicating that stem cell progenitors reside mainly in crypts. Unlike LGR5, Sox9 expression has also been previously shown at low levels in enteroendocrine and tuft cells, consistent with the low SOX9 expression found in the villi of our dogs [33, 34].

**Table 2:** RNA in situ hybridization (ISH) probe details

Gene	RNAscope® Probe	Catalog no.	Accession no.	Target region
Leucine-rich repeat-containing G protein-coupled Receptor 5	Cl-LGR5	405651	XM_846738.2	517–1506
Sry-related high-mobility group box 9	Cl-SOX9	502551	NM_001002978.1	2–1536
Ephrin receptor B2	Cl-EPHB2	515361	XM_005617823.2	180–1757
Intestinal-specific alkaline phosphatase	Cl-ALPI	515391	XM_534605.6	783–1791
Frizzled class receptor 5	Cl-FZD5	515371	XM_003640189.3	301–1514
neurogenin 3	Cl-NEUROG3	515271	XM _546140.1	2–575
Canine interleukin 17 (IL-17)	Cl-IL17A	502541	NM_001165878	2–1178
Canine beta defensin 103 (CBD 103)	Cl-CBD103	515411	NM_001129980	2–389
Canine cathelicidin (CATH)	Cl-CAMP	515401	NM_001003359.1	2–607
Doublecortin-like kinase 1 (Dclk1)	Cl-DCLK1	515311	XM_014107394.1	401–1547
Prostaglandin-receptor (EP4R)	Cl-PTER4	499011	NM_001003054.1	535–1706

Since lysozyme staining for Paneth cells was negative, we next explored expression of other Paneth cell markers responsible for maintaining the ISC niche in dogs [28]. Both ephrin type-B receptor 2 (EPHB2) and Frizzled-5 (FZD5) have a role in canonical Wnt signaling and are also expressed in mouse Paneth cells [35–37]. Similar to LGR5, EPHB2, another marker of intestinal stemness as well as Paneth cells [35], was mainly expressed in the crypts of enteroids and jejunal tissues as compared to the villus compartment (*P* = 0.0001). FZD5 was found both in crypts and, to a lesser degree, in the villi of enteroids and jejunal tissues, with very little expression observed in the villus tips (*P* = 0.001) (Fig. 5b, c). Given the putative role of Paneth cells in innate immunity and host defense, we also determined expression of various pro-inflammatory cytokines and antimicrobial peptides produced by Paneth cells, including interleukin-17 (IL-17), beta defensin 103 (CBD 103), and cathelicidin (CATH), using canine-specific probes [38]. CBD103 and CATH, both antimicrobial peptides, were observed both in whole tissue and in enteroids, whereas IL-17 was found only in whole tissue lamina propria, but not in the epithelium (Fig. 5d).

We further characterized markers for absorptive enterocyte and enteroendocrine cells with RNAscope target probes for canine Intestinal Alkaline Phosphatase (ALP) and Neurogenin-3 (NeuroG3), respectively [39, 40]. Intestinal alkaline phosphatase (ALP), a brush border enzyme marker for differentiated epithelia, was observed to be more highly expressed in the villi (surface epithelia) of enteroids and jejunal tissues versus cryptal epithelium (*P* = 0.0001) (Fig. 5b, c). NeuroG3, a marker for enteroendocrine cells and indirect marker for Paneth cells [40], was dispersed throughout all regions of crypt and villi (Fig. 5b, c), with the full-thickness tissue jejunal crypt epithelia showing increased expression as compared to the enteroid crypt epithelia (*P* = 0.0296).

Tuft cells, named for their microvilli projections, were identified by a canine Doublecortin-Like Kinase 1 (Dclk1) probe [41]. Tuft cells function as chemosensory cells that initiate type 2 immune responses to helminth parasites [42]. Indeed, under normal conditions the tuft cell population is low, but tuft cell numbers are greatly increased by parasite infection [42]. Dclk1, a marker for tuft cells, was uniformly expressed in crypt and villi of both enteroids and full-thickness tissue (Fig. 5d).

**Fig. 6:**
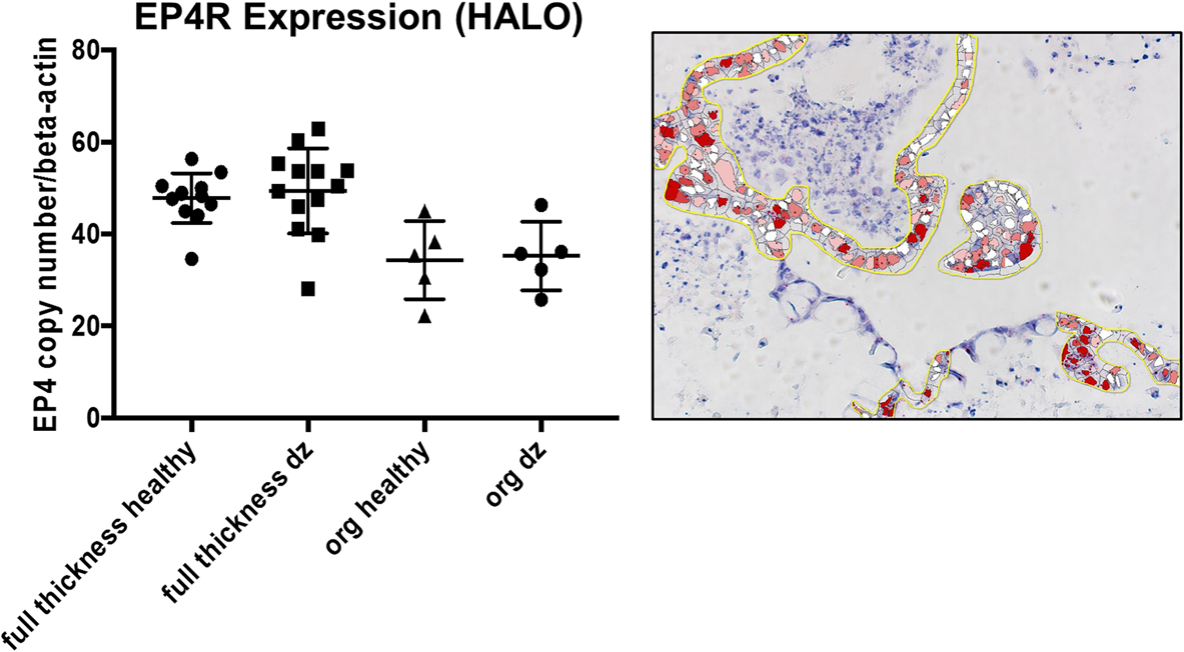
Prostaglandin E2 receptor-4 expression is similar between tissue and enteroids from healthy and IBD dogs. Representative RNA-ISH image illustrates how the Prostaglandin E2 receptor-4 (EP4R) staining was marked for quantification using Halo software. Box and whisker plot compares the EP4R expression among biopsy tissues (full thickness) and enteroids (org) obtained from both healthy and IBD (dz) dogs. Scoring of at least *n* = 5 images per condition

Finally, we examined the expression of the EP4 prostaglandin-receptor (EP4R), a receptor that has been implicated in the pathogenesis of IBD and is a target of anti-inflammatory and analgesic drugs including nonsteroidal anti-inflammatory drugs (NSAIDS), COX-2 inhibitors, and the piprant class, which are selective EP4R inhibitors. We characterized the expression of EP4R in whole intestinal tissues and enteroids of healthy and diseased dogs to determine the role of EP4R in IBD pathophysiology. There was no difference in epithelial expression of EP4R between biopsies of dogs with IBD and enteroids from IBD dogs (*p* = 0.37) (Fig. 6), indicating that canine enteroids are accurately model EP4R expression in whole tissue. Furthermore, there was no statistically significant difference in EP4R between enteroids of healthy dogs versus enteroids of IBD dogs (*p* = 0.84), nor in the biopsies of healthy dogs vs. dogs diagnosed with IBD (*p* = 0.37) (Fig. 6).

### Functional assays in canine enteroids

**Fig. 7:**
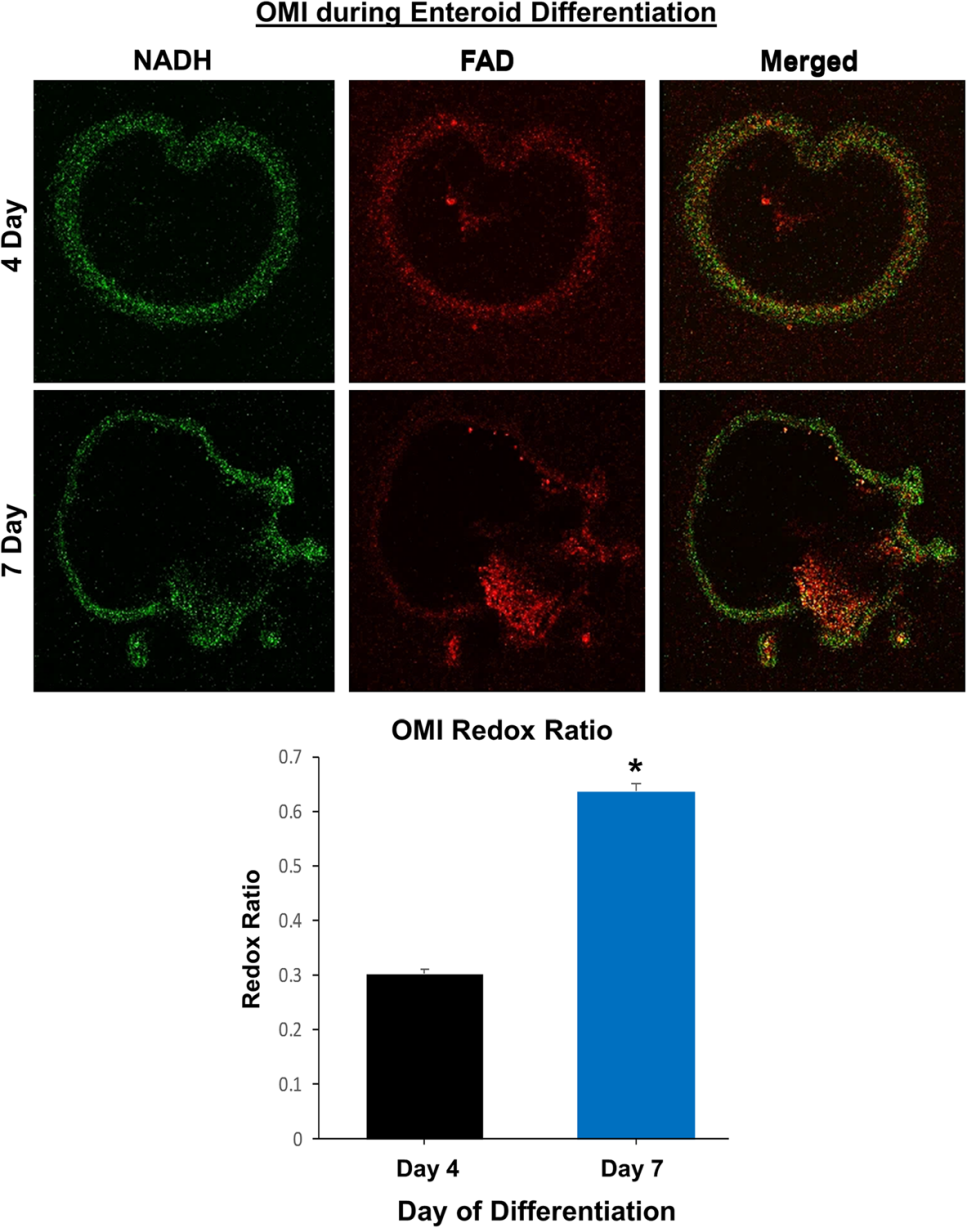
Optical metabolic imaging (OMI) reveals metabolic differences during differentiation of canine enteroids. Representative fluorescent images from 4- and 7-day-old enteroids are shown. The green image indicates the NADH measurement whereas the red image indicates the FAD, with the merged images on the right. The graph shows the optical redox ratio of 4-day-old versus 7-day-old enteroids (0.303 + 0.008 versus 0.637 + 0.013, respectively, for mean + SEM). *n* = 15 organoids per condition

Optical metabolic imaging (OMI), a new technology consisting of fluorescence imaging using a multi-photon microscope, has high resolution and sensitivity to accurately measure cellular metabolic changes, unlike other imaging techniques such as FDG-PET [43]. We used OMI to characterize the metabolic differences between enteroids during differentiation by calculating the optical redox ratio per enteroid. As expected, the optical redox ratio of 7-day-old enteroids was approximately twice that of 4-day-old enteroids, indicating a lower redox state of the early enteroids versus the older and more differentiated enteroids (Fig. 7).

**Fig. 8:**
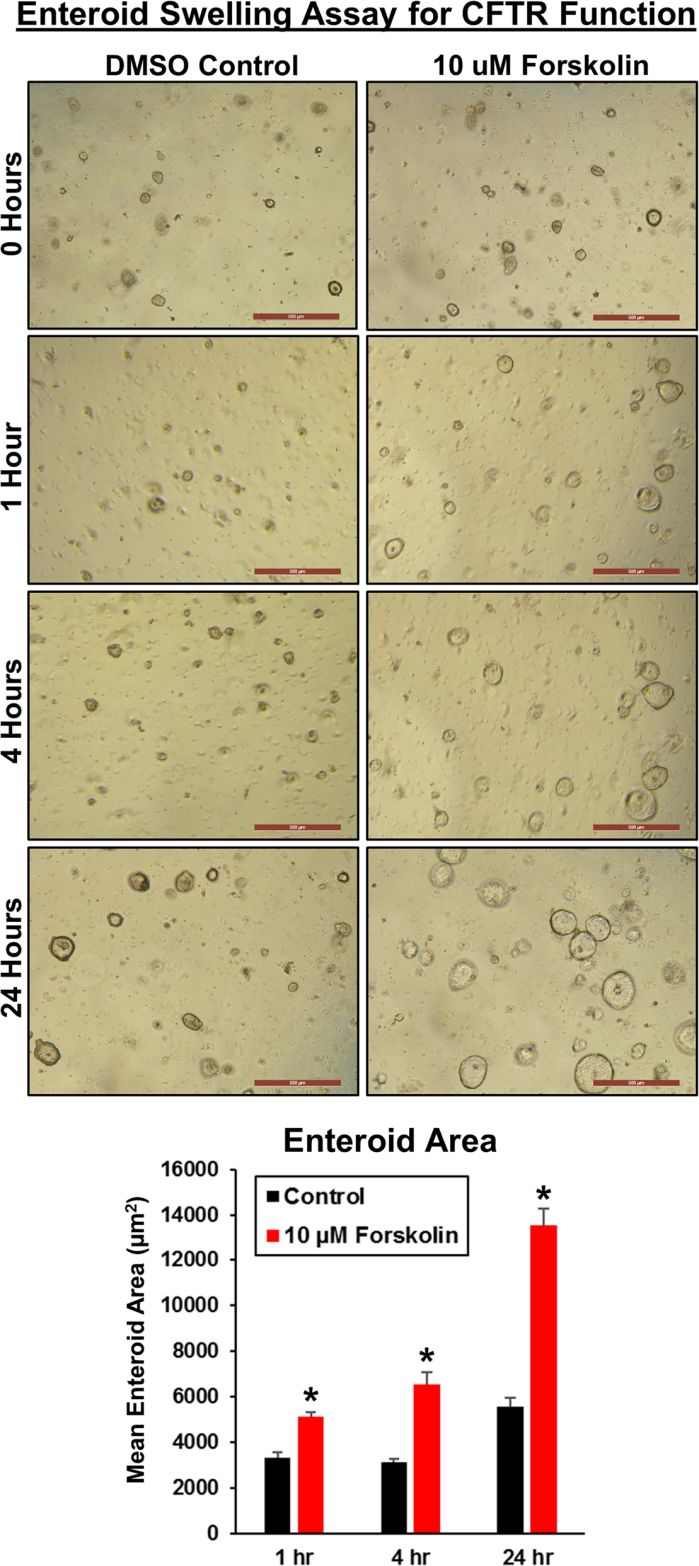
Forskolin induces swelling of canine enteroids in a time-dependent manner, indicating presence of functional CFTR. Enteroids were passaged and seeded in Matrigel into 24-well plates. After 2 days, enteroids were incubated in media containing vehicle control (DMSO) or 10 μM forskolin. Representative images of enteroids were taken after 0, 1, 4, and 24 h, at × 5 magnification. Graph of mean area of enteroids (15–25 per field) from *n* = 12 fields per condition as determined by ImageJ (mean + SEM; *p* < 0.05 vs. control for each time)

We next used forskolin, a cyclic adenosine monophosphate (cAMP) agonist, to activate the cystic fibrosis transmembrane conductance regulator (CFTR) chloride channels in intestinal epithelial cells to induce swelling of canine enteroids [13]. As activation of the CFTR chloride channel depends on intracellular cAMP levels, forskolin induces enteroid swelling by increasing water flux into the lumen of the enteroids. This assay can therefore be used as an indirect measure of CFTR function in enteroids [13]. Similar to human intestinal colonoids, incubation with 10 μM forskolin stimulated the swelling of day 2 jejunal enteroids from healthy dogs [13]. Forskolin-induced enteroid swelling was observed after 1, 4, and 24 h, and the swelling increased in a time-dependent manner (Fig. 8), confirming functionality of CFTR in the canine enteroids, similar to intact tissue.

**Fig. 9:**
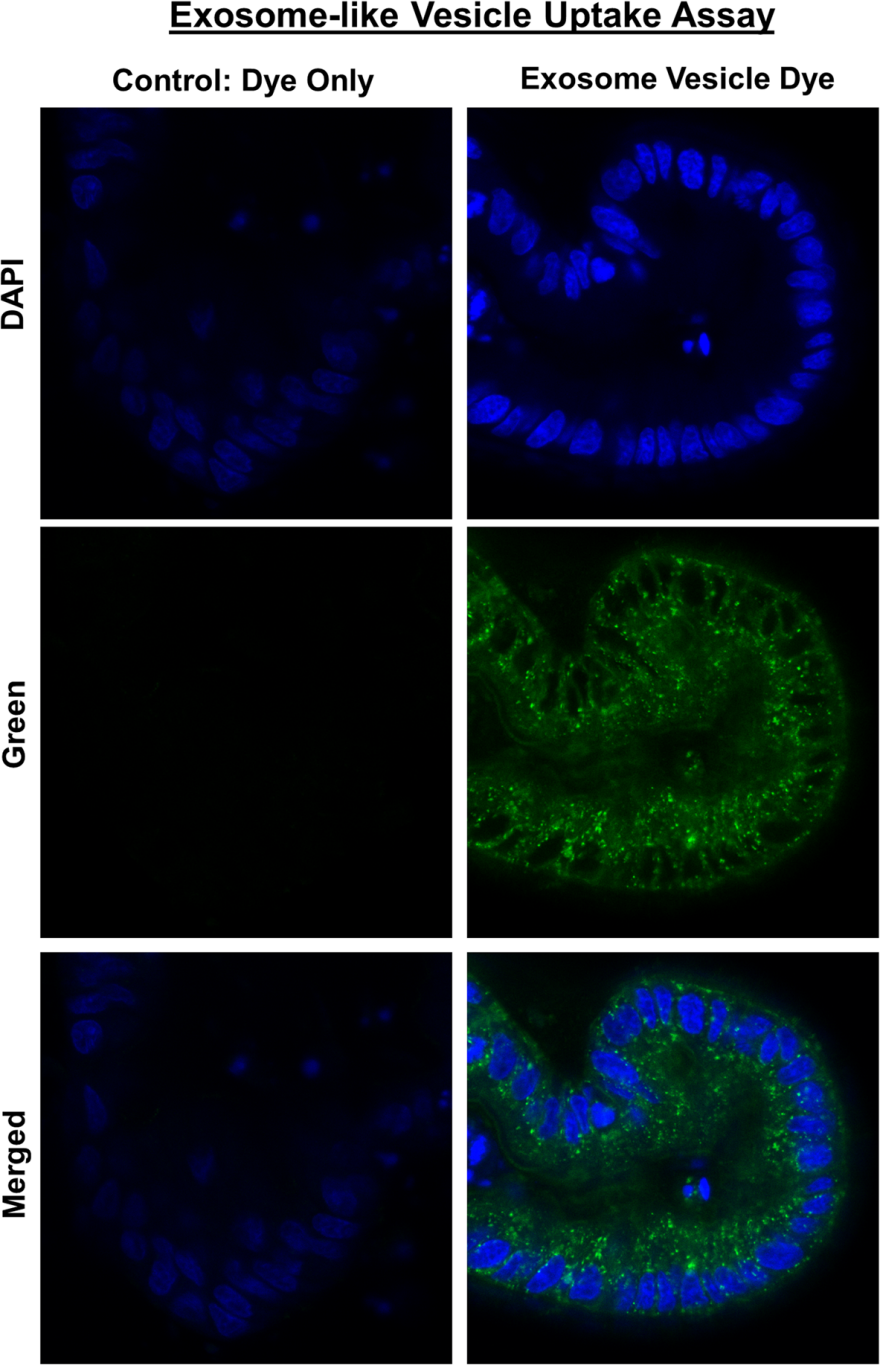
Canine enteroids uptake exosome-like vesicles secreted from the parasite *Ascaris suum*. Representative confocal microscope images taken after 24-h incubation with control fluorophore alone (green fluorescent PKH67) or exosome-like vesicles (EV) labeled with PKH67 green fluorescent dye. Enteroids were counterstained with the nuclear marker DAPI (blue fluorescence). Only exosome+ PKH67 group demonstrated green fluorescence within epithelial cells and within the organoid lumen. The merged image shows the intracytoplasmic localization of the PKH67 green fluorescent dye labeled EV obtained from *Ascaris suum*

We next investigated whether exosome-like vesicles from *Ascaris suum* nematodes could be phagocytized by enteroids. Enteroids incubated for 24 h with exosome-like vesicles labeled with PKH67 dye demonstrated green fluorescent-labeled exosomes within epithelial cells and within the enteroid lumen (Fig. 9). In contrast, enteroids treated with PKH67 dye alone had only DAPI nuclear staining (Fig. 9). These data indicated functional uptake of exosomes with transport of vesicles through the epithelial cells and into the enteroid lumen within 24 h.

## Discussion

### Canine enteroids and colonoids as a translational model for GI research

In our study, we were able to successfully culture crypt epithelium obtained from different canine intestinal regions, including the duodenum, jejunum, ileum, and colon as well as intestinal tumors, using an adapted protocol for isolating human crypt cells [25]. We investigated cultivation of organoids (enteroids and colonoids) from both whole tissue and endoscopically-derived intestinal specimens obtained from healthy dogs and dogs with spontaneous GI diseases, including IBD and colorectal cancer. Jejunal enteroids were characterized in depth using IHC, RNA-ISH, morphometric, and TEM ultrastructure. Further, we demonstrated the functional utility of canine enteroids by performing OMI, a CFTR function assay and visualization of the active uptake of parasitic extracellular vesicles obtained from *Ascaris suum*. Therefore, these studies form the foundation for the development of canine enteroids as an ex vivo large animal model for translational GI research.

A previous study derived terminal ileum enteroids from two dogs as well as enteroids from other companion and farm animal species [44]. Our work, using biopsy samples from over 40 dogs and four different intestinal locations, established the conditions for long-term organoid culture from multiple physiological conditions. We are able to culture not only canine enteroids from large whole intestine tissue sections similar to the previous report [44], but also enteroids/colonoids from much smaller (3 mm diameter) intestinal endoscopic biopsy samples from live dogs using a relatively non-invasive procedure, greatly expanding the pool of potential donors to dogs with various GI enteropathies, especially veterinary client patients that may be undergoing endoscopy for disease diagnosis or analysis.

Canine enteroids and colonoids are a powerful translational and mechanistic tool for identifying molecular targets and therapeutic cures for chronic intestinal diseases such as IBD and CRC. As enteroids and colonoids are composed of epithelial cells alone, they can be used as a platform for the screening of drug candidates that target the epithelial components of intestinal diseases [4]. In addition, canine enteroids and colonoids complement animal-based GI toxicology studies and potentially reduce the number of animals needed for in vivo studies. Canine enteroids and colonoids are also useful for targeted therapy and personalized medicine, as these organoids are derived from individual dogs with different genotypes, environmental risk factors, and drug sensitivity profiles. Combining canine enteroids and colonoids with microfluidic chips will help in the development of precision medicine and the study of interactions between the gut and microbiome. In addition, orthotopic transplantation of canine enteroids or colonoids has the potential to repair or replace damaged or dysfunctional epithelial tissues for regenerative medicine therapies in diseases including IBD, cystic fibrosis, and tuft cell enteropathy. Among the large animal models used in translational GI research, the dog is particularly relevant as it shares similar environmental, genomic, anatomical, intestinal physiologic, and pathologic features with humans [45–48]. Although pigs are by far the most popular large animal model used for transplantation and cardiovascular studies, there are several limitations of the porcine model, including the absence of naturally occurring GI diseases analogous to humans, the high cost for husbandry, the technical difficulties in handling pigs, and issues in collecting biological specimens, specifically with regard to obtaining endoscopic biopsies of the GI tract [4].

Undoubtedly, the use of mouse stem cells and organoids revolutionized the study of developmental biology; however, murine models often lack key clinical signs or pathological changes representative of complex human intestinal diseases, such as IBD [49]. In fact, there are growing concerns from the NIH, pharmaceutical industry, and biomedical researchers about the ability of murine models to accurately represent human phenotypes, as illustrated by recurrent failures in translating drug safety and efficacy data from murine models to human patients [50–52]. As such, the success of therapeutic approaches based on intestinal stem cells requires refinement in animal models, including animals that have organs comparable in size and physiology to those of humans [2]. Therefore, it is imperative to develop innovative and robust large animal systems to support intestinal stem cell translational research and the development of new therapeutic discoveries for human digestive diseases [49].

### Canine enteroids model canine intestinal tissue phenotypes

While we obtained enteroids and colonoids from healthy and diseased dogs, we characterized enteroids from healthy jejunum in greater depth due to their importance in nutrient and drug absorption [53]. Our data indicate canine jejunal 3D enteroids are capable of recapitulating the anatomical and physiologic features of native jejunal tissue. Unlike whole intestinal tissues that contain epithelial, mesenchymal, and immune cells, adult ISC-derived enteroids and colonoids from mice and humans consist only of epithelial cell populations [30, 31]. Epithelial markers (keratin, chromogranin A, and PAS) were present in both canine enteroids and whole jejunal tissues, whereas mesenchymal (vimentin and actin) and immune cell (c-kit and CD3 T cell) markers were found only in whole jejunal tissues (Table 1), indicating that canine enteroids also consist of epithelial populations without other cell types.

Epithelial tight junctions are critical for maintaining the intestinal barrier as well as for a multitude of physiological functions of intestinal epithelial cells, including nutrient absorption and drug transport across the mucosa [54]. In this study, we utilized TEM to assess the structural integrity of epithelial tight junctions as it provides easier characterization of ultrastructural changes compared with other methods, such as IHC [55]. Our TEM studies confirmed that both jejunal tissues and enteroids contain adherens junctions (AJ), tight junctions (TJ), and desmosomes. These proteins are specialized membrane structures that mediate cell-to-cell contact and provide the structural basis for interactions between adjacent intestinal epithelial cells [56, 57]. We found that the spacing or gaps in these structures between cells decreased throughout differentiation in the enteroids, creating a less porous intestinal barrier after 6 to 9 days of culture.

The identification of stem and progenitor cell populations in enteroids has proven critical to understanding whether stemness of whole jejunal tissue is retained in enteroids, as well as the impact of in vitro culture and maintenance on intestinal stem cells in enteroids. We used a canine-specific LGR5 RNAscope probe to identify crypt base columnar stem cells, since LGR5-positive adult ISCs are essential to develop the characteristic crypt-villus enteroid structures in the absence of a non-epithelial cell niche [30]. LGR5-positive adult ISCs were mainly observed in the crypt region of both enteroids and jejunal tissues, which is similar to reports of LGR5-positive adult ISCs in mice and humans [30, 31]. In contrast, Sox9, another marker of ISCs, was found to be expressed both in the intestinal crypts and, to a lesser extent, in the villus compartments of normal dogs. Although Sox9 is primarily expressed in ISCs and transit-amplifying progenitor cells, it is also found in other epithelial populations, such as enteroendocrine and Tuft cells [32–34]. Therefore, the distribution pattern of SOX9 mRNA in enteroids may not only represent ISC stem cells, but likely indicates the presence of other secretory lineage epithelial cells. In addition to Sox9, Dclk1 expression in enteroids and intestinal tissues confirms the presence of tuft cells [41].

Positive IHC staining for Alcian Blue/PAS and Chromogranin A for cells in jejunal enteroids indicate that canine ISC can differentiate into specialized epithelial cells including goblet and enteroendocrine cells, as in jejunal tissues. TEM ultrastructure characterization supported the IHC findings and confirmed the presence of enteroendocrine and goblet cells in 3D enteroids. We further investigated the presence of differentiated epithelial cells in enteroids using RNA-ISH and found expression for the absorptive enterocyte marker ALP and the enteroendocrine/secretory lineage cell marker NeuroG3. In addition, TEM revealed microvilli in the canine enteroids and jejunal tissues, and the number and lengths of microvilli increased during enteroid differentiation. Microvilli are cellular membrane protrusions of absorptive enterocytes containing different populations of brush border enzymes involved in absorption, secretion, and cellular adhesion [26]. Our TEM results showing microvilli support our RNA-ISH data of expression of the brush border enzyme ALP, and both microvilli and ALP expression indicate the presence of differentiated absorptive enterocytes.

Paneth cells are highly specialized epithelial cells located in the base of the crypts of Lieberkühn of the small intestine of some species including mice and humans and are involved in the production of growth factors necessary for ISC function [38]. Since Paneth cells are reported to be absent in some mammalian species, including the dog, functionally equivalent cells remained to be identified in this species. Consistent with this, lysozyme IHC staining and IL-17 RNA-ISH was negative in our canine enteroids as well as the epithelium of full-thickness jejunum. We therefore investigated EPHB2 and FZD5 expression in canine enteroids as both receptors are involved in maintenance of intestinal stemness through canonical Wnt signaling and are expressed in murine Paneth cells [35, 36, 58]. In addition, the Wnt receptor Fzd5 is required for Paneth cell differentiation and localization to the crypt base in mice [36]. In our study, EPHB2 and FZD5 were expressed in both enteroids and jejunum tissues, suggesting a similarly important role of Wnt signaling in the canine species. In addition to their role in supporting the growth of ISCs, Paneth cells also play an important role in innate immune defense by producing antimicrobial peptides, including CBD 103 and CATH [38]. Our findings of CBD 103 and CATH expression, in addition to EPHB2 and FZD5, support the presence of a functional array of Paneth-like cells in dogs. Altogether, these findings demonstrate that our canine 3D enteroid model system accurately reproduces the anatomical and phenotypical features of canine small intestines, including a broad spectrum of fully differentiated epithelial cells.

Canine enteroids are also a useful in vitro model for diseased small intestine tissue. PGE2 is a key mediator of inflammation that acts through EP1, EP2, EP3, and EP4 receptors and has been implicated in the development and progression of various cancers [58, 59]. Newer NSAIDs (piprant class) have been developed by targeting EP4R specifically, which should help reduce common GI side effects of NSAID drugs [60]. Our results show that EP4R was expressed in the epithelium of full-thickness jejunum and enteroids of healthy and diseased dogs and confirm the utility of canine enteroids to investigate the effects of NSAIDs on EP4R expression in GI tissues. These data also lay the foundation for development of drug testing assays for the piprant class of drugs including grapiprant, a selective EP4R antagonist, using canine enteroids from healthy dogs and dogs with intestinal inflammation, including IBD.

### Functional uses and translational applications of canine intestinal enteroids

In our study, OMI was able to distinguish and quantitate the cellular metabolism between young growing enteroids (day 4) and differentiated late enteroids (day 7) using optical redox ratio calculations. OMI has been used previously to determine metabolic activity of various types of tumor organoids in response to drug treatment, but this technique may also prove useful for personalized medicine using canine intestinal and CRC organoids [43]. In addition, we further explored canine enteroid function by examining forskolin-induced swelling of enteroids as a measure of CFTR function. CFTR is the chloride channel mutated in cystic fibrosis, and loss of CFTR function leads to progressive decreases in lung function, pancreatic exocrine dysfunction, and intestinal obstruction or constipation [61]. We found that incubation with forskolin doubled the area of canine jejunal enteroids after 4 h, indicating the presence of functional CFTR chloride channels, similar to human intestinal colonoids [13]. Thus, the forskolin-swelling CFTR function assay with canine enteroids has translational applications in drug screening, especially given that dogs have naturally occurring CFTR mutations similar to humans [13, 62].

Finally, to evaluate the absorptive and barrier functionality of enteroids, we developed a 3D canine enteroid uptake assay using parasitic EVs produced by intestinal helminths. We used EVs from the *Ascaris suum* parasite because of its zoonotic nature and significance in veterinary medicine [63]. EVs are known to elicit host immune responses because of their rich miRNA and bioactive protein contents [64, 65], which are hypothesized to induce tolerance towards the helminths in the host organism. Therefore, given the uptake of EVs by our canine enteroids, our 3D enteroid model may be useful to study host-pathogen interactions for parasites that are important in both animal and human disease.

### Canine intestinal crypt isolation, culture, and maintenance

Unlike the previous report on canine enteroid culture using collagenase digestion for crypt epithelial isolation [44], we employed a cold EDTA chelation method. The EDTA chelation technique is the method of choice for crypt isolation, allowing maximum purity of crypt epithelium and minimum contamination of other cell types [25]. We were able to not only culture canine enteroids from large whole intestine tissue sections similar to the previous report [44], but also from much smaller (3 mm diameter) intestinal endoscopic biopsy samples. In our study, we used 5–10 times higher EDTA concentration than reported for the mouse crypt epithelial isolation protocol, but similar to that used for humans [25, 66]. In addition, canine ileal tissue samples required a greater EDTA concentration with longer incubation periods for optimal digestion, similar to the treatment of human ileal tissue samples [25]. Variations between mammalian species in requirements for EDTA concentrations and incubation times for release of ISCs from the different intestinal segments are influenced by species differences in length, numbers and size of villi [66].

For our studies, we used human ISC culture media containing Wnt-3a for cultivating canine intestinal enteroids and colonoids, as it resulted in better colony forming efficiency (CFE) as compared to mouse ISC culture media, which does not contain Wnt-3a (data not shown). Our observation is consistent with the previous report showing that Wnt-3a, R-spondin-3, and Noggin are required to propagate intestinal enteroids from both farm and companion small animals, including the dog [44]. Additionally, we used Y-27632, an inhibitor of ROCK, and CHIR99021, an inhibitor of GSK-3, for the first 2 days of culture to enhance the initial survival and facilitate long-term propagation of enteroids/colonoids as well as to prevent dissociation-induced apoptosis (anoikis). Moreover, the addition of the ROCK inhibitor, Y-27632, into the initial ISC culture medium improved CFE of organoids in our preliminary experiments (data not shown).

## Conclusions

In summary, we have developed and maintained long-term canine enteroid and colonoid 3D models using whole tissue and biopsy samples from over 40 healthy dogs and dogs with chronic enteropathies. Our enteroid system complements existing preclinical animal models and provides a translational platform for drug permeability, efficacy, and safety screening. Finally, the tools developed and validated in our lab for the canine 3D intestinal enteroid and colonoid systems include a comprehensive set of reagents, probes, and functional assays, which will serve as a foundation for using the dog as a translational model for precision and regenerative medicine.

## Methods

### Animals

The collection and analysis of intestinal tissues and biopsies from healthy dogs and dogs with IBD was approved by the Iowa State University (ISU) Institutional Animal Care and Use Committee (IACUC protocols: 4-17-8504-K, 9-17-8605-K, 3-17-8489-K, and 12-04-5791-K).

### Collection of full-thickness and endoscopically obtained intestinal tissues

For full-thickness intestinal tissues, a 5–10 cm length segment of the proximal jejunum of healthy dogs was collected within 30 min of euthanasia. The tissue was next cut open longitudinally and luminal contents removed by forceps. Tissue was immediately placed in wash medium (PBS with 2 mM *N*-acetylcysteine) and vigorously shaken 10–15 times, repeating washes four times, in order to remove excess mucus, residual luminal contents, and other debris [67]. After washing, cleansed tissues were transferred to culture media without growth factor (CMGF− consisting of Advanced DMEM/F12 (Fisher) supplemented with 2 mM GlutaMax-1 (Fisher Scientific), 10 mM Hepes and 100 μg/mL Primocin (InvivoGen)) and incubated on ice. GI endoscopy biopsy forceps (Olympus America) were used to collect mucosa tissue samples from the whole tissue segment. Similarly, 10–15 duodenal, ileal, and colonic endoscopic biopsies were obtained by forceps from healthy or IBD dogs under general anesthesia by veterinary gastroenterologists. For clinical research cases, collected biopsies were placed in CMGF− medium on ice and subjected to mechanical cleansing as described above.

### Crypt cell isolation and enrichment from intestinal tissues and biopsies

Epithelial crypts containing adult ISC were isolated and enriched from intestinal tissue and tumor samples following a standard procedure adapted from a published human organoid culture protocol [25]. Both whole tissue samples and endoscopic biopsies were cut into small pieces (1–2 mm thickness) with a scalpel and washed six times using complete chelating solution (1X CCS), consisting of 5X CCS (2.49 g Na_2_HPO_4_-2H_2_O, 2.7 g KH_2_PO_4_, 14 g NaCl, 0.3 g KCl, 37.5 g Sucrose, and 25 g D-Sorbitol in 500 mL Milli-Q H_2_O) diluted 1:5 in Milli-Q H_2_O and supplemented with 0.52 μM DTT. Pipettes and conical tubes were pre-wetted with 1% bovine serum albumin (BSA) throughout the procedure to prevent adherence of the crypt epithelium to tubes and pipettes, thereby minimizing loss of crypt cells [67]. Then, tissues were incubated with 1X CCS containing EDTA (20–30 mM) for 45 to 75 min at 4 °C on 20 , 24 rpm mixer/rocker (Fisher). Length of time for digestion varied depending on collection site, with ileal mucosa requiring 75 min of incubation and higher EDTA concentrations (30 mM) versus other intestinal segments. The incubation time was determined by the absence of crypts in tissue fragments, apparent as “holes” and the presence of cellular, dense spheroids under phase contrast microscope (Additional file 2: Figure S1) [67]. After EDTA chelation, release of the cryptal epithelium was augmented by trituration and/or mild vortexing in CCS. Additional trituration and/or mild vortexing was carried out after adding fetal bovine serum (FBS; Atlanta Biologicals) to maximize crypt release. After tissue fragments settled to the bottom of the tube, the crypt containing supernatant was transferred to a new conical tube, then centrifuged at 150*g* at 4 °C for 5 min. After centrifugation, the pellet was washed with 10 mL CMGF− medium, containing Advanced DMEM/F12 (Gibco) with 1:100 Glutamax (Fisher), 200 mM HEPES (Fisher), and 100 μg/ml Primocin (InvivoGen), and centrifuged at 70*g* at 4 °C for 5 min. The crypt pellet was then resuspended in 2 mL CMGF− medium, and the approximate number of crypts isolated was calculated using a hemocytometer.

### ISC subculture and organoid maintenance

Approximately 400–800 crypts per 10–20 biopsy samples were obtained depending on the GI collection site. Among the four different sites, colonic biopsies yielded the greatest number of ISCs (~ 800 crypts), the duodenum yielded the least (~ 400 crypts), and the jejunum and ileum yielded an intermediate number of crypts (~ 600 crypts). An estimated 50–100 crypts were seeded per well in 30 μL of Matrigel (Corning® Matrigel® Growth Factor Reduced (GFR) Basement Membrane Matrix) into a 24-well plate format and incubated at 37 °C for 10 min [66]. Complete medium with ISC growth factors (CMGF+), containing CMGF− medium with 1X B27 (Fisher), 1X N2 (Fisher), 1 mM *N*-acetylcysteine (Sigma-Aldrich), 50 ng/mL EGF (PeproTech), 100 ng/mL Noggin (PeproTech), 500 ng/mL R-spondin-1 (PeproTech), 100 ng/mL Wnt 3a (PeproTech), 10 nM Gastrin (Sigma-Aldrich), 10 mM Nicotinamide (Sigma-Aldrich), 5 mM A83-01 (TGFβ type I receptor inhibitor; Tocris), 50 μM SB202190 (P38 inhibitor; Sigma-Aldrich), and 8% FBS (Atlanta Biologicals), supplemented with 10 μM rho-associated kinase inhibitor (ROCKi) Y-27632 (StemGent) and 2.5 μM glycogen synthase kinase 3β (GSK3β) inhibitor CHIR99021 (StemGent), was added, and the plate was incubated at 37 °C. The CMGF+ medium with ROCK and GSK3β inhibitors was used for the first 2 days of ISC culture to enhance ISC survival and prevent apoptosis [68]. The concentration of these growth factors and the requirement of Wnt3a was optimized in our pilot experiments. CHIR99021, an inhibitor of GSK-3, in combination with Y-27632, an inhibitor of ROCK, was only added temporarily to the media for the first 2 days after isolation of intestinal crypts for enteroid/colonoid to culture and is then removed. The short-term addition of the GSK-3 inhibitor, CHIR99021, enhanced the initial survival and facilitated long-term propagation of enteroids/colonoids, while including Wnt3a in the media long-term improved colony forming efficiency and was required for enteroid/colonoid survival beyond three passages. Removal of the Rock and GSK3 inhibitors from the media after the first 2 days of culture then improved differentiation of the canine enteroids/colonoids. A similar media containing Wnt3a, R-spondin, CHIR99021, and other compounds is utilized for equine enteroids, although the timing of specific mediums differs from our canine studies [69]. The enteroid and colonoid cultures were replenished with CMGF+ medium every 2 days. After 6–8 days, enteroids and colonoids were completely differentiated, showing a luminal compartment, crypt epithelium, and villi-like structures along with exfoliation of denuded epithelia into the lumen (Fig. 1). Therefore, the ideal passage expansion of canine enteroids and colonoids is carried out every 5–7 days, just prior to epithelial shedding. For passaging, enteroids and colonoids contained within Matrigel were mechanically disrupted using cold CMGF− medium and either subjected to trituration with a needle syringe (23 g ¾″) or, alternatively, incubated with warm TrypLE Express at 37 °C for ~ 10 min followed by addition of CMGF− medium to stop enzyme activity. The dissociated enteroids and colonoids were pelleted by centrifuging at 4 °C × 100*g* for 5 min and resuspended in Matrigel and cultured as described above. Representative phase contrast photomicrographs of at least *n* = 15 (healthy dogs), *n* = 7 (IBD dogs), and *n* = 4 (tumors) images of enteroids/colonoids are shown for each location.

### Cryopreservation of enteroids and colonoids

For cryopreservation and bio-archiving, recovery cell freezing media (Invitrogen) was used to facilitate recovery of enteroids and colonoids post-thaw [68]. However, we also tested 90% FBS with 10% DMSO freezing media for some enteroids and colonoids and did not find a difference between this freezing media and the recovery cell freezing media in quality or quantity of enteroids and colonoids recovered after freezing with either preservation media (Additional file 3: Figure S2). Approximately 50–200 organoids were frozen per vial, and the amount of vials cryopreserved per dog and location varied depending on experimental need and use as well as the number of passages of organoid expansion (Additional file 1: Table S1). We opted to preserve whole canine enteroids and colonoids in liquid nitrogen using the commercial recovery cell freezing media to maintain consistency in cryopreservation. For recovery, enteroids and colonoids were thawed in cold CMGF− medium, centrifuged at 100*g* for 5 min at 4 °C, resuspended in thawed Matrigel, and seeded into 24-well plates as above.

### Tissue and enteroid fixation and processing for histopathology and TEM

Representative segments of jejunum tissue and enteroids were incubated in 10% formalin and then stored in 70% ethanol for histopathology (H&E), immunohistochemistry (IHC), and RNA-ISH. Fixed tissue and enteroids were paraffin embedded and cut in 4-μm sections onto glass slides. Representative images from at least *n* = 15 enteroids per condition are shown for H&E. For TEM, enteroids and tissue were initially placed in a fixative solution composed of 2% paraformaldehyde/3% glutaraldehyde/0.1 M cacodylate with pH 7.2, followed by 1% osmium tetroxide post fixative. Thick (1 μm) and ultrathin (50–100 nm) sections were cut by microtome, collected on copper grids and observed under a JEOL 2100 200 kV STEM. Representative images are presented from at least *n* = 10 enteroids per condition.

### IHC characterization of enteroids and tissues

IHC was performed using established protocols [70–73]. Briefly, sections were deparaffinized and rehydrated using an automated system with manual antigen retrieval and blocking steps. Primary antibody, biotinylated secondary antibody, horseradish peroxidase-streptavidin, and NovaRed staining were applied on the section in a sequential order followed by counterstaining. Markers used in IHC were pan-Keratin, Chromogranin A, Vimentin, Actin, c-Kit, T cell, PAS, and Lysozyme. Presence and absence of these markers was evaluated using light microscopy and the cell types determined by morphology, location and staining, and pictures were taken (at least *n* = 20 organoids per condition). The list of markers and the antibody source, dilution and incubation details are given in Additional file 4: Table S2.

### RNA-ISH characterization of jejunal enteroids and tissues

RNA In Situ Hybridization (ISH) was performed using the RNAscope 2.5 High Definition (Red) kit per the manufacturer’s protocol (Advanced Cell Diagnostics, Newark, CA, USA). Briefly, enteroids and tissue sections were deparaffinized, boiled in target retrieval solution, and incubated in protease buffer. Then sections were hybridized with specific oligonucleotide probes for cell surface markers of intestinal stem cells, epithelial differentiation, and maturation, and then the respective mRNA was serially amplified [29]. The details of the probe and sequences are given in Table 2. After amplification, signal was detected with RED-B and RED-A, and hematoxylin used as counterstain. Representative microscope images from *n* = 15 or more enteroids per condition are shown. The expression of mRNA markers was calculated from *n* = 6 images per condition (at least *n* = 5 images per condition for EP4R) using the singleplex semi-quantitative scoring criteria adapted from ACD Pharma Assay Services data analysis for the intensity of staining (< 1 dot/10 cells is no staining; 1–3 dots/cell is +; 4–9 dots/cell is ++; 10–15 dots/cell is +++; > 15 dots/cell is ++++). The staining intensity between different mucosal regions including crypt and villi was compared.

### Optical metabolic imaging (OMI) of canine enteroids

Day 4 and 7 enteroids from healthy dogs underwent OMI as one measure of functional activity, using a two-photon FLIM microscope incorporating time correlated single photon counting [43]. NADH and FAD were imaged at two-photon excitation wavelengths of 750 nm and 890 nm, respectively, with emission collected between 400–480 nm and 500–600 nm, using a × 20/1.15 NA water immersion objective. A minimum of 15 image volumes were captured within each sample. To quantify the effect of different age enteroids (4-day-old versus 7-day-old) on cellular metabolism of the enteroids as a whole, we estimated the optical redox ratio of the intensities of cellular cofactors NADH and FAD, as described by Walsh et al. [43]. Optical redox ratio was calculated as NADH intensity divided by FAD intensity per organoid and was the average of *n* = 15 samples per condition.

### CFTR functional assay using canine enteroids

Enteroids were passaged and seeded in Matrigel into 24-well plates as above. After 2 days, enteroids were incubated in CMGF+ media containing vehicle control (DMSO) or the indicated treatments [13]. Representative images of enteroids were taken after 0, 1, 4, and 24 h at × 5 magnification on an inverted microscope using the Leica Application Suite (LAS) software. Six wells and two fields per well were used for each condition. The average enteroid area for each field (15–25 enteroids per field) was determined using ImageJ software, and then mean enteroid area calculated for *n* = 12 fields per condition.

### Exosome-like vesicle uptake assay using canine enteroids

*Ascaris suum* female adult nematode worms were obtained from JBS Swift and Co. pork processing plant (Marshalltown, IA, USA) and cultured using the *Ascaris* Ringer’s solution (ARS): 13.14 mM NaCl, 9.47 mMCaCl2, 7.83 mM MgCl2, 12.09 mM Tris, 99.96 mM sodium acetate, 19.64 mM KCl, pH 7.8 supplemented with 5 mM glucose, 10,000 units pen/strep, 10 μg/mL ciprofloxacin, and 0.25 μg/mL amphotericin [63]. Culture media was collected between 24 and 48 h and passed through a 0.22-μM filter (EMD Millipore, USA). Extracellular vesicles (EVs) secreted by the helminths were purified by ultracentrifugation protocol as previously described [64]. Briefly, the medium containing EVs was centrifuged at 4 °C × 120,000*g* for 90 min (Becman SW32Ti rotor); then the EV pellet was resuspended in DPBS (Thermofisher, USA), transferred to a new tube, and centrifuged at 4 °C × 154,000*g* for 2 h (Beckman TLA55 rotor). The EV pellet was resuspended with DPBS and stored at − 80 °C. The protein concentration of EVs was determined using a Qubit Fluorometer (Thermofisher, USA). Purified EVs were stained with PKH67 Fluorescent dye (Sigma, USA) as per the manufacturer’s protocol. In brief, 10-μg EVs were resuspended with the diluents solution and then stained with PKH67 dye at room temperature for 5 min. The labeling reaction was stopped by mixing with an equal volume of FBS. Then, the EVs were pelleted by centrifuging at 4 °C×154,000*g* for 2 h (Beckman TLA55 rotor). Pellets were resuspended with 1000 μL DPBS (Thermofisher, USA). An equal volume of CMGF+ medium was mixed with EVs labeled with PKH67 dye. As a positive control, an equal volume of CMGF+ medium was mixed with PKH67 dye alone. Ileal enteroids from healthy dogs were cultivated on 8-well chamber slides, and on day 3, enteroids were incubated with CMGF+ medium containing control, dye only control, or labeled exosome-like vesicles for 24 h at 37 °C. Then, enteroids were washed three times with PBS and fixed in 4% paraformaldehyde (Sigma-Aldrich). After fixation, enteroids were washed with PBS, counterstained with DAPI, and visualized at × 40 magnification using a Leica TCS SP5 X Confocal/multi-photon microscope system (Leica Microsystems Inc., Buffalo Grove, IL, USA).

### Statistical analysis

Data were analyzed using graph pad prism version 7 and R version 3.5. RNA-ISH semi-quantitative data were analyzed using Kruskal-Wallis Statistical test for multiple comparisons of groups. Dunn’s post hoc testing was used wherever specific comparisons between two groups were needed. Student’s *t* test was used to analyze significance for other experiments where appropriate. Statistical significance was set at *p* < 0.05.

## Additional files

Additional file 1: **Table S1.** Details of dogs used for isolation, propagation, and preservation of canine 3D intestinal organoids. (PDF 96 kb)

Additional file 2: **Table S2.** IHC antibody, dilution, and incubation time. (PDF 93 kb)

Additional file 3: **Figure S1.** Appearance of holes after EDTA incubation indicates release of crypts from intestinal tissue. Representative image of colon tissue after EDTA incubation, showing apparent holes and dense cellular spheroids by phase contrast microscope (× 20 magnification). (PPTX 5174 kb)

Additional file 4: **Figure S2.** Comparison of medium for cryopreservation. Representative images of fully differentiated canine enteroids by phase contrast microscope (× 5 magnification). There was no discernible difference in quality or quantity of organoids recovered after freezing with either commercial cell freezing media (Invitrogen) or 90% FBS with 10% DMSO. (PPTX 964 kb)

## References

[1] Perlman RL (2016). Mouse models of human disease: an evolutionary perspective. Evol Med Public Health.

[2] Ziegler A, Gonzalez L, Blikslager A (2016). Large animal models: the key to translational discovery in digestive disease research. Cell Mol Gastr Hepatol.

[3] Wong SK, Chin KY, Suhaimi FH, Fairus A, Ima-Nirwana S (2016). Animal models of metabolic syndrome: a review. Nutr Metab.

[4] Mochel JP, Jergens AE, Kingsbury D, Kim HJ, Martín MG, Allenspach K (2017). Intestinal stem cells to advance drug development, precision, and regenerative medicine: a paradigm shift in translational research. AAPS J.

[5] Ericsson AC, Crim MJ, Franklin CL (2013). A brief history of animal modeling. Mo Med.

[6] Franco NH (2013). Animal experiments in biomedical research: a historical perspective. Animals..

[7] Schaefer K, Rensing S, Hillen H, Burkhardt JE, Germann PG (2016). Is science the only driver in species selection? An internal study to evaluate compound requirements in the minipig compared to the dog in preclinical studies. Toxicol Pathol.

[8] Ranga A, Gjorevski N, Lutolf MP (2014). Drug discovery through stem cell-based organoid models. Adv Drug Deliv Rev.

[9] Lancaster MA, Renner M, Martin C-A, Wenzel D, Bicknell LS, Hurles ME (2013). Cerebral organoids model human brain development and microcephaly. Nature..

[10] McCracken KW, Catá EM, Crawford CM, Sinagoga KL, Schumacher M, Rockich BE (2014). Modelling human development and disease in pluripotent stem-cell-derived gastric organoids. Nature..

[11] Greggio C, De Franceschi F, Figueiredo-Larsen M, Gobaa S, Ranga A, Semb H (2013). Artificial three-dimensional niches deconstruct pancreas development in vitro. Dev Camb Engl.

[12] Múnera JO, Wells JM (2017). Generation of gastrointestinal organoids from human pluripotent stem cells. Methods Mol Biol.

[13] Dekkers JF, Wiegerinck CL, de Jonge HR, Bronsveld I, Janssens HM, Winter-de Groot KM (2013). A functional CFTR assay using primary cystic fibrosis intestinal organoids. Nat Med.

[14] Ciancanelli MJ, Huang SXL, Luthra P, Garner H, Itan Y, Volpi S (2015). Infectious disease. Life-threatening influenza and impaired interferon amplification in human IRF7 deficiency. Science..

[15] Qian X, Nguyen HN, Song MM, Hadiono C, Ogden SC, Hammack C (2016). Brain-region-specific organoids using mini-bioreactors for modeling ZIKV exposure. Cell..

[16] Fukuda M, Mizutani T, Mochizuki W, Matsumoto T, Nozaki K, Sakamaki Y (2014). Small intestinal stem cell identity is maintained with functional Paneth cells in heterotopically grafted epithelium onto the colon. Genes Dev.

[17] Clevers H (2016). Modeling development and disease with organoids. Cell..

[18] Lancaster MA, Knoblich JA (2014). Organogenesis in a dish: modeling development and disease using organoid technologies. Science.

[19] Dutta D, Heo I, Clevers H (2017). Disease modeling in stem cell-derived 3D organoid systems. Trends Mol Med.

[20] Fatehullah A, Tan SH, Barker N (2016). Organoids as an in vitro model of human development and disease. Nat Cell Biol.

[21] Sun D, Yu LX, Hussain MA, Wall DA, Smith RL, Amidon GL (2004). In vitro testing of drug absorption for drug 'developability' assessment: forming an interface between in vitro preclinical data and clinical outcome. Curr Opin Drug Discov Devel.

[22] Hussein SM, Batada NN, Vuoristo S, Ching RW, Autio R, Närvä E (2011). Copy number variation and selection during reprogramming to pluripotency. Nature..

[23] Laurent LC, Ulitsky I, Slavin I, Tran H, Schork A, Morey R (2011). Dynamic changes in the copy number of pluripotency and cell proliferation genes in human ESCs and iPSCs during reprogramming and time in culture. Cell Stem Cell.

[24] Blutt SE, Crawford SE, Ramani S, Zou WY, Estes MK (2018). Engineered human gastrointestinal cultures to study the microbiome and infectious diseases. Cell Mol Gastroenterol Hepatol..

[25] Saxena K, Blutt SE, Ettayebi K, Zeng XL, Broughman JR, Crawford SE (2015). Human intestinal enteroids: a new model to study human rotavirus infection, host restriction, and pathophysiology. J Virol.

[26] Grant CN, Mojica SG, Sala FG, Hill JR, Levin DE, Speer AL, Barthel ER, Shimada H, Zachos NC, Grikscheit TC (2015). Human and mouse tissue-engineered small intestine both demonstrate digestive and absorptive function. Am J Physiol Gastrointest Liver Physiol.

[27] Hudson L (2002). Keratins as markers of epithelial cells. Meth Mol Biol.

[28] Gelberg H (2014). Comparative anatomy, physiology, and mechanisms of disease production of the esophagus, stomach, and small intestine. Toxicol Pathol.

[29] Yokoyama N, Ohta H, Yamazaki J, Kagawa Y, Ichii O, Khoirun N (2017). Localization of toll-like receptor (TLR) 2 and TLR4 mRNA in the colorectal mucosa of miniature dachshunds with inflammatory colorectal polyps. Comp Pathol.

[30] Sato T, Vries RG, Snippert HJ, van de Wetering M, Barker N, Stange DE (2009). Single Lgr5 stem cells build crypt-villus structures in vitro without a mesenchymal niche. Nature..

[31] Sato T, Stange DE, Ferrante M, Vries RG, Van Es JH, Van den Brink S (2011). Long-term expansion of epithelial organoids from human colon, adenoma, adenocarcinoma, and Barrett’s epithelium. Gastroenterology.

[32] Ramalingam S, Daughtridge GW, Johnston MJ, Gracz AD, Magness ST (2012). Distinct levels of Sox9 expression mark colon epithelial stem cells that form colonoids in culture. Am J Physiol Gastrointest Liver Physiol.

[33] Formeister EJ, Sionas AL, Lorance DK, Barkley CL, Lee GH (2009). Distinct SOX9 levels differentially mark stem/progenitor populations and enteroendocrine cells of the small intestine epithelium. Am J Physiol Gastrointest Liver Physiol.

[34] Gracz AD, Ramalingam S, Magness ST (2010). Sox9 expression marks a subset of CD24-expressing small intestine epithelial stem cells that form organoids in vitro. Am J Physiol Gastrointest Liver Physiol.

[35] Nalapareddy K, Nattamai KJ, Kumar RS, Karns R, Wikenheiser-Brokamp KA, Sampson LL (2017). Canonical Wnt signaling ameliorates aging of intestinal stem cells. Cell Rep.

[36] van Es JH, Jay P, Gregorieff A, van Gijn ME, Jonkheer S, Hatzis P (2005). Wnt signalling induces maturation of Paneth cells in intestinal crypts. Nat Cell Biol.

[37] Noah TK, Donahue B, Shroyer NF (2011). Intestinal development and differentiation. Exp Cell Res.

[38] Clevers HC, Bevins CL (2013). Paneth cells: maestros of the small intestinal crypts. Annu Rev Physiol.

[39] Goldberg RF, Austen WG, Zhang X, Munene G, Mostafa G, Biswas S (2008). Intestinal alkaline phosphatase is a gut mucosal defense factor maintained by enteral nutrition. PNAS..

[40] Schonhoff SE, Giel-Moloney M, Leiter AB (2004). Neurogenin 3-expressing progenitor cells in the gastrointestinal tract differentiate into both endocrine and non-endocrine cell types. Dev Biol.

[41] Middelhoff M, Westphalen CB, Hayakawa Y, Yan KS, Gershon MD, Wang TC (2017). Dclk1-expressing tuft cells: critical modulators of the intestinal niche. Am J Physiol Gastrointest Liver Physiol.

[42] Gerbe F, Jay P (2017). Intestinal tuft cells: epithelial sentinels linking luminal cues to the immune system. Mucosal Immunol.

[43] Walsh AJ, Cook RS, Sanders ME, Arteaga CL, Skala MC (2016). Drug response in organoids generated from frozen primary tumor tissues. Sci Rep.

[44] Powell RH, Behnke MS (2017). WRN conditioned media is sufficient for in vitro propagation of intestinal organoids from large farm and small companion animals. Biol Open.

[45] Kararli TT (1995). Comparison of the gastrointestinal anatomy, physiology, and biochemistry of humans and commonly used laboratory animals. Biopharm Drug Dispos.

[46] Vázquez-Baeza Y, Hyde ER, Suchodolski JS, Knight R (2016). Dog and human inflammatory bowel disease rely on overlapping yet distinct dysbiosis networks. Nat Microbiol.

[47] Cerquetella M, Spaterna A, Laus F, Tesei B, Rossi G, Antonelli E (2010). Inflammatory bowel disease in the dog: differences and similarities with humans. World J Gastroenterol.

[48] Xenoulis PG, Palculict B, Allenspach K, Steiner JM, Van House AM, Suchodolski JS (2008). Molecular-phylogenetic characterization of microbial communities imbalances in the small intestine of dogs with IBD. FEMS Microbiol Ecol.

[49] Harding J, Roberts RM, Mirochnitchenko O (2013). Large animal models for stem cell therapy. Stem Cell Res Ther.

[50] Waring MJ, Arrowsmith J, Leach AR, Leeson PD, Mandrell S, Owen RM (2015). An analysis of the attrition of drug candidates from four major pharmaceutical companies. Nat Rev Drug Discov.

[51] Cibelli J, Emborg ME, Prockop DJ, Roberts M, Schatten G, Rao M (2013). Strategies for improving animal models for regenerative medicine. Cell Stem Cell.

[52] Nakamura T, Sato T (2018). Advancing intestinal organoid technology toward regenerative medicine. Cell Mol Gastroenterol Hepatol.

[53] Spiller RC (1994). Intestinal absorptive function. Gut..

[54] Anderson JM, Van Itallie CM (2009). Physiology and function of the tight junction. Cold Spring Harb Perspect Biol.

[55] Du Plessis J, Vanheel H, Janssen CE, Roos L, Slavik T (2013). Activated intestinal macrophages in patients with cirrhosis release NO and IL-6 that may disrupt intestinal barrier function. J Hepatol.

[56] Hartsock A, Nelson WJ (2008). Adherens and tight junctions: structure, function and connections to the actin cytoskeleton. Biochim Biophys Acta.

[57] Kowalczyk AP, Green KJ (2013). Structure, function, and regulation of desmosomes. Prog Mol Biol Transl Sci.

[58] Schuijers J, Clevers H (2012). Adult mammalian stem cells: the role of Wnt, Lgr5 and R-spondins. EMBO J.

[59] Ricciotti E, FitzGerald GA (2011). Prostaglandins and inflammation. Arterioscler Thromb Vasc Biol.

[60] Woodward DF, Jones RL, Narumiya S (2011). International Union of Basic and Clinical Pharmacology. LXXXIII: classification of prostanoid receptors, updating 15 years of progress. Pharmacol Rev.

[61] Cutting GR (2015). Cystic fibrosis genetics: from molecular understanding to clinical application. Nat Rev Genet.

[62] Spadafora D, Hawkins EC, Murphy KE, Clark LA, Ballard ST (2010). Naturally occurring mutations in the canine CFTR gene. Physiol Genomics.

[63] Nejsum P, Betson M, Bendall RP, Thamsborg SM, Stothard JR (2012). Assessing the zoonotic potential of Ascaris suum and Trichuris suis: looking to the future from an analysis of the past. J Helminthol.

[64] Harischandra Hiruni, Yuan Wang, Loghry Hannah J., Zamanian Mostafa, Kimber Michael J. (2018). Profiling extracellular vesicle release by the filarial nematode Brugia malayi reveals sex-specific differences in cargo and a sensitivity to ivermectin. PLOS Neglected Tropical Diseases.

[65] Simpson RJ, Kalra H, Mathivanan S (2012). ExoCarta as a resource for exosomal research. Journal of extracellular vesicles.

[66] Mahe MM, Aihara E, Schumacher MA, Zavros Y, Montrose MH, Helmrath MA, Sato T, Shroyer NF (2013). Establishment of gastrointestinal epithelial organoids. Curr Protoc Mouse Biol.

[67] Kingsbury DD, Mochel JP, Atherly T, Chandra LC, Phillips RL, Hostetter J, Wannemuehler MJ, Jergens A, Allenspach K (2018). Mo1059: comparison of endoscopically (Egd/Colo) procured enteroids and colonoids from Normal dogs and dogs with naturally occurring chronic enteropathies (IBD). Gastroenterology..

[68] Han SH, Shim S, Kim MJ, Shin HY, Jang WS, Lee SJ (2017). Long-term culture-induced phenotypic difference and efficient cryopreservation of small intestinal organoids by treatment timing of Rho kinase inhibitor. World J Gastroenterol.

[69] Stewart AS, Freund JM, Gonzalez LM (2018). Advanced three-dimensional culture of equine intestinal epithelial stem cells. Equine Vet J.

[70] Vos JH, van den Ingh TS, Ramaekers FC, de Neijs M, van Mil FN, Ivanyi D. Keratin and vimentin distribution patterns in the epithelial structures of the canine anal region. Anat Rec. 1992;234(3):391–8. 10.1002/ar.1092340309 1280011

[71] Grube D, Yoshie S (1989). Immunohistochemistry of chromogranin A and B, and secretogranin II in the canine endocrine pancreas. Arch Histol Cytol.

[72] Noland EL, Kiupel M (2018). Coexpression of CD3 and CD20 in canine enteropathy-associated T-cell lymphoma. Vet Pathol.

[73] Magi GE, Mariotti F, Berardi S, Piccinini A, Vullo C, Palumbo Piccionello A, Rossi G (2018). Loss of alpha-smooth muscle actin expression associated with chronic intestinal pseudo-obstruction in a young miniature bull terrier. Acta Vet Scand.

