# Supplementary material for: Derivation of adult canine intestinal organoids for translational research in gastroenterology
Source: BMC Biol. 2019 Apr 11;17:33. doi: 10.1186/s12915-019-0652-6 (PMC6460554; doi:10.1186/s12915-019-0652-6)
Supplement: Supplementary file 1 — Table S1. Details of dogs used for isolation, propagation, and preservation of canine 3D intestinal organoids. (PDF 96 kb) [file 12915_2019_652_MOESM1_ESM.pdf]

**Additional file 1: Table S1.** Details of dogs used for isolation, propagation and preservation of canine 3D intestinal organoids.

| | | | | | # of vials archived | | | |
|---|---|---|---|---|---|---|---|---|
| | Type of Dog | # of Dogs | Tissue type | Tissue site/location | Duodenum | Jejunum | Ileum | Colon |
| 1 | Healthy | 20 | Whole tissue | 15 dogs: Jejunum | | 78 | | |
| | | | | 5 dogs: Duodenum, Ileum, Colon | 2 | | 14 | 15 |
| 2 | Healthy Nutrition Study Dogs: | | | | | | | |
| 2.a | Pre-treatment | 8 | Biopsy | Duodenum, Ileum, Colon | 23 | | 30 | 26 |
| 2.b | Post-treatment | 8 | Biopsy | Duodenum, Ileum, Colon | 39 | | 56 | 58 |
| 3 | IBD Dogs | 9 | Biopsy | Duodenum, Ileum, Colon | 8 | | 13 | 14 |
| 4 | Lysosyme storage mutation | 1 | Whole tissue | Duodenum, Ileum, Colon | | | 4 | 4 |
| 5 | Tumor | 2 | Biopsy | | 5 | | 2 | 2 |

Definitions: Biopsy, endoscopically obtained biopsy tissue; IBD, inflammatory bowel disease; Nutrition Dogs, healthy dogs before and after diet treatment.
