# Supplementary material for: Derivation of adult canine intestinal organoids for translational research in gastroenterology
Source: BMC Biol. 2019 Apr 11;17:33. doi: 10.1186/s12915-019-0652-6 (PMC6460554; doi:10.1186/s12915-019-0652-6)
Supplement: Supplementary file 2 — Table S2. IHC antibody, dilution, and incubation time. (PDF 93 kb) [file 12915_2019_652_MOESM2_ESM.pdf]

Additional file 2: Table S2. IHC antibody, dilution and incubation time.

Primary Ab	Source	Pretreatment	Dilution	Incubation	Secondary Ab
Keratin (alt)	Dako	Tris-EDTA pH 9.0, 20 min steam	1:100	90 min	goat anti-mouse (Multi-Link)
T-cell (CD3)	Dako	Tris-EDTA pH 9.0, 20 min steam	1:100	90 min	goat anti-rabbit Multi-Link)
Vimentin	Dako	Citra pH 6.0, 5 min	1:500	30 min	goat anti-mouse (Multi-Link)
Chromogranin A	Santa Cruz	Tris-EDTA pH 9.0, 20 min steam	1:100	60 min	Vector, Gt. ABC kit
CD117, c-kit	Dako	Tris-EDTA pH 9.0, 20 min steam	1:500	120 min	goat anti-rabbit (Multi-Link)
Actin (sm)	BioGenix	None	1:200	30 min	goat anti-mouse (Multi-Link)
Lysozyme	Dako	Tris-EDTA pH 9.0, 20 min steam	1:100	90 min	goat anti-rabbit Multi-Link)
