# Supplementary material for: Derivation of adult canine intestinal organoids for translational research in gastroenterology
Source: BMC Biol. 2019 Apr 11;17:33. doi: 10.1186/s12915-019-0652-6 (PMC6460554; doi:10.1186/s12915-019-0652-6)
Supplement: Supplementary file 3 — Figure S1. Appearance of holes after EDTA incubation indicates release of crypts from intestinal tissue. Representative image of colon tissue after EDTA incubation, showing apparent holes and dense cellular spheroids by phase contrast microscope (× 20 magnification). (PPTX 5174 kb) [file 12915_2019_652_MOESM3_ESM.pptx]

## Slide 1
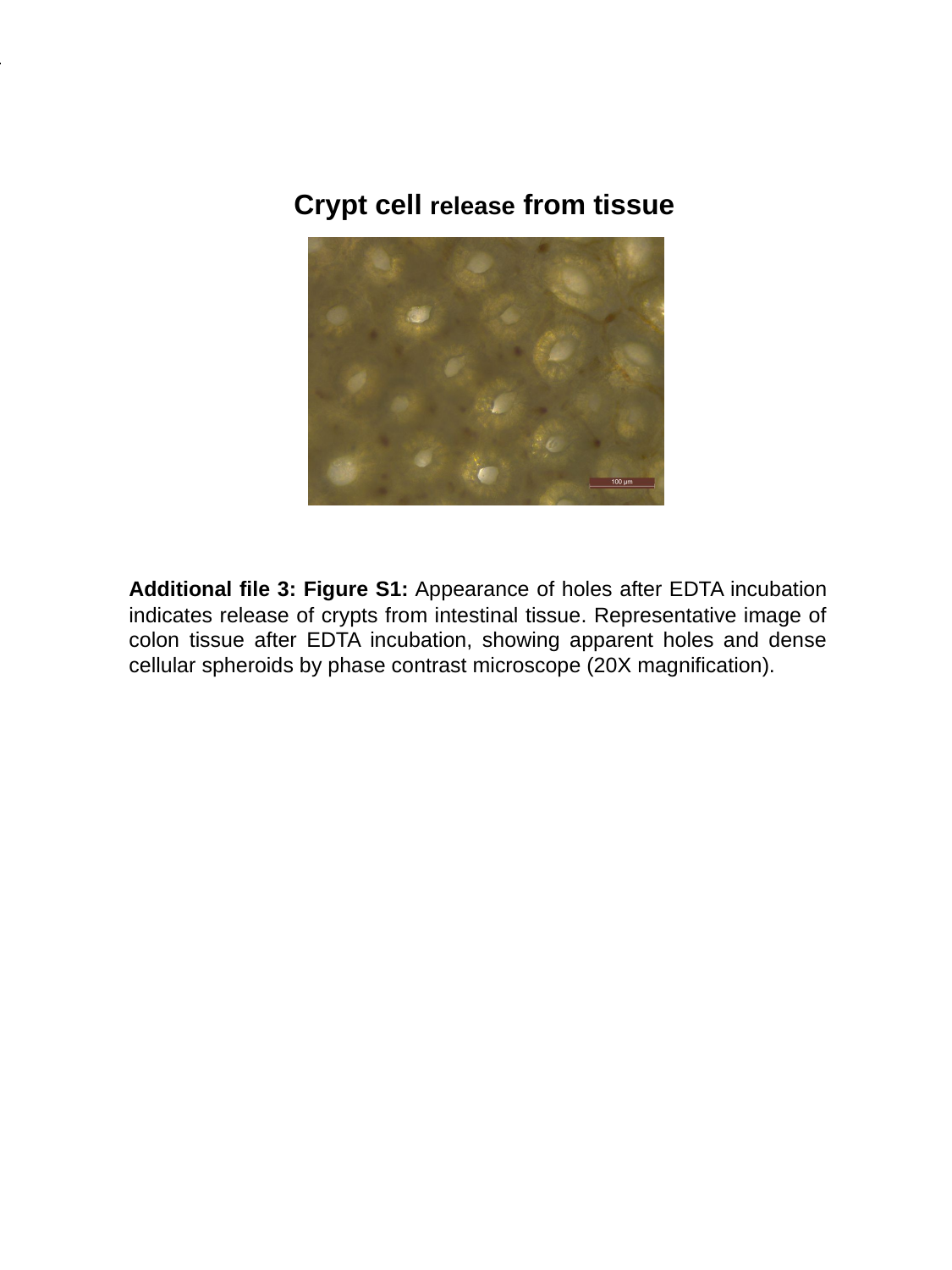

Crypt cell release from tissue
Additional file 3: Figure S1: Appearance of holes after EDTA incubation indicates release of crypts from intestinal tissue. Representative image of colon tissue after EDTA incubation, showing apparent holes and dense cellular spheroids by phase contrast microscope (20X magnification).
