# Supplementary material for: Derivation of adult canine intestinal organoids for translational research in gastroenterology
Source: BMC Biol. 2019 Apr 11;17:33. doi: 10.1186/s12915-019-0652-6 (PMC6460554; doi:10.1186/s12915-019-0652-6)
Supplement: Supplementary file 4 — Figure S2. Comparison of medium for cryopreservation. Representative images of fully differentiated canine enteroids by phase contrast microscope (× 5 magnification). There was no discernible difference in quality or quantity of organoids recovered after freezing with either commercial cell freezing media (Invitrogen) or 90% FBS with 10% DMSO. (PPTX 964 kb) [file 12915_2019_652_MOESM4_ESM.pptx]

## Slide 1
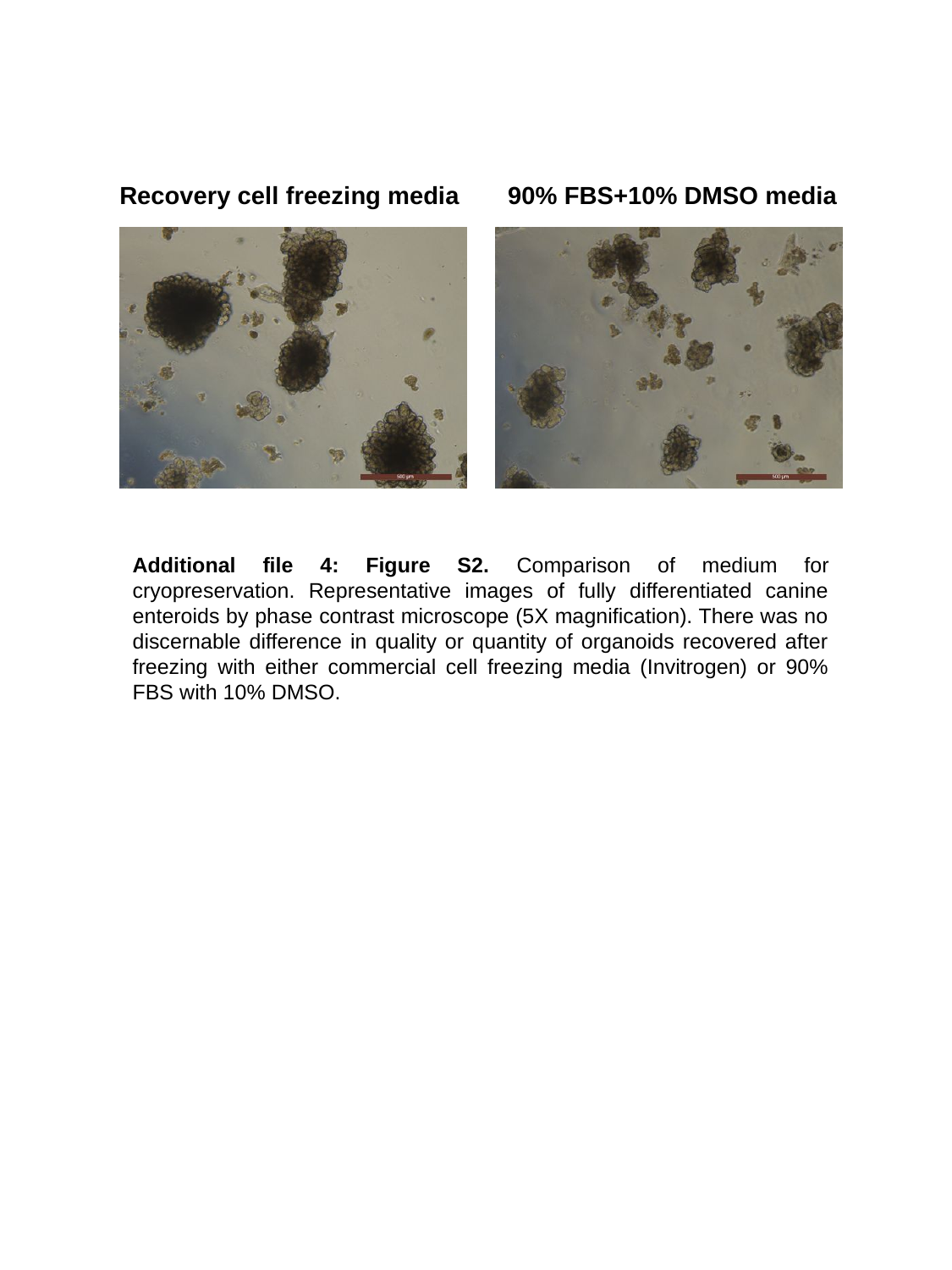

Recovery cell freezing media 90% FBS+10% DMSO media
Additional file 4: Figure S2. Comparison of medium for cryopreservation. Representative images of fully differentiated canine enteroids by phase contrast microscope (5X magnification). There was no discernable difference in quality or quantity of organoids recovered after freezing with either commercial cell freezing media (Invitrogen) or 90% FBS with 10% DMSO.
